# Complete Mapping of DNA‐Protein Interactions at the Single‐Molecule Level

**DOI:** 10.1002/advs.202101383

**Published:** 2021-10-05

**Authors:** Wenzhe Liu, Jie Li, Yongping Xu, Dongbao Yin, Xin Zhu, Huanyan Fu, Xiaodong Su, Xuefeng Guo

**Affiliations:** ^1^ State Key Laboratory for Structural Chemistry of Unstable and Stable Species Beijing National Laboratory for Molecular Sciences National Biomedical Imaging Center College of Chemistry and Molecular Engineering Peking University Beijing 100871 P. R. China; ^2^ Shenzhen Bay Laboratory Shenzhen 518132 P. R. China; ^3^ State Key Laboratory of Protein and Plant Gene Research Biomedical Pioneering Innovation Center (BIOPIC) Peking University Beijing 100871 P. R. China; ^4^ Center of Single‐Molecule Sciences Frontiers Science Center for New Organic Matter Institute of Modern Optics College of Electronic Information and Optical Engineering Nankai University 38 Tongyan Road, Jinnan District Tianjin 300350 P. R. China

**Keywords:** DNA–protein interactions, real‐time detections, single‐molecule studies, SiNW FETs

## Abstract

DNA–protein interaction plays an essential role in the storage, expression, and regulation of genetic information. A 1D/3D facilitated diffusion mechanism has been proposed to explain the extraordinarily rapid rate of DNA‐binding protein (DBP) searching for cognate sequence along DNA and further studied by single‐molecule experiments. However, direct observation of the detailed chronological protein searching image is still a formidable challenge. Here, for the first time, a single‐molecule electrical monitoring technique is utilized to realize label‐free detection of the DBP–DNA interaction process based on high‐gain silicon nanowire field‐effect transistors (SiNW FETs). The whole binding process of WRKY domain and DNA has been visualized with high sensitivity and single‐base resolution. Impressively, the swinging of hydrogen bonds between amino acid residues and bases in DNA induce the dynamic collective motion of DBP–DNA. This in situ, label‐free electrical detection platform provides a practical experimental methodology for dynamic studies of various biomolecules.

## Graphical abstract

A single‐molecule electrical detection platform, based on a silicon nanowire field‐effect transistors (SiNW FETs) nanocircuit, is applied to real‐time monitor the complete interaction process of WRKY1N and DNA with single‐base‐pair resolution. A long‐last collective motion correlated to the specific recognition site is observed at the single‐molecule level, providing a novel technique with single‐molecule/single‐event sensitivity to decipher the comprehensive mechanism of biomolecule interactions.

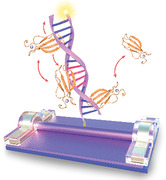

## 1 Introduction

DNA‐binding proteins (DBPs) need to recognize specific DNA sequences to ensure that target genes are expressed under a certain time‐space condition at an appropriate intensity, particularly in the regulation of transcription.^[^ ^1^, ^2^, ^3^, ^4^ ^]^ The discussion about the mechanism of DBPs’ rapid searching for cognate DNA sequences has been raised since the discovery that lac repressor can search their target sequences at a much faster rate than the limit of the 3D diffusion.^[^ ^5^ ^]^ In the following investigations,^[^ ^6^, ^7^, ^8^, ^9^, ^10^ ^]^ a 1D/3D facilitated searching mechanism is developed to divide DBP and DNA interaction patterns into three types. DBPs initially 3D diffuse to and bind with the DNA molecules non‐specifically, then relocate to the target sequence site rapidly by a 1D sliding and hopping along DNA strands or translocating between contacting fragments of DNA molecules.^[^ ^3^, ^11^ ^]^ Although ensemble experiments verify the 1D/3D facilitated searching mechanism,^[^ ^9^, ^12^ ^]^ many important details of the interaction process are still unclear due to the limitation of the ensemble methods, such as the differentiation and the heterogeneity among sliding, hopping, or intersegment transfer.^[^ ^13^ ^]^ In contrast, single‐molecule techniques make it possible to investigate individual molecules and provide much more detailed information of transient states, rare events, and heterogeneous behaviors, which are generally covered by the ensemble average.^[^ ^3^, ^14^, ^15^, ^16^, ^17^ ^]^

**Figure 1:**
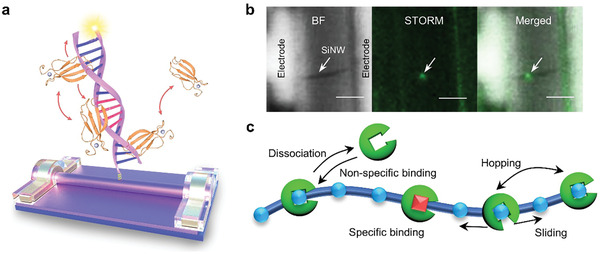
Schematic structure and characterization of a single‐DNA modified SiNW FET device. a) Schematic of a single‐DNA modified SiNW FET device. b) Bright field (BF) image (left), STORM image (medium), and the merged image (right) of a single‐DNA modified device after treated by a 1 nmol L^−1^ Cy3‐labeled DNA solution. Scale bar: 5 µm. c) Schematic of the interaction between WRKY1N protein and W‐box DNA. Green circles represent protein, blue circles and red squares represent non‐specific and cognate sequences.

However, there are still tough challenges to acquire a whole vision of the DBP–DNA interaction dynamic process in experimental researches due to the limitation of spatio‐temporal resolution (10−50 nm, 10−100 ms) of single‐molecule technologies,^[^ ^14^, ^15^, ^16^, ^17^, ^18^, ^19^, ^20^, ^21^, ^22^, ^23^, ^24^, ^25^ ^]^ and the insufficient time scale for dynamic simulation also limits theoretical studies.^[^ ^13^, ^26^, ^27^, ^28^ ^]^ In this work, we utilize a single‐biomolecule electrical detection platform based on high‐gain SiNW FETs to realize label‐free detection (**Figure** **1**a and Figure S1, Supporting Information).^[^ ^29^, ^30^, ^31^ ^]^ WRKY family proteins as important transcriptional factors in plants play an important role for signal response, stress control and disease resistance. Remarkably, we monitored every detail of the whole binding process of the W‐box DNA and the WRKY1N protein, the N‐terminal WRKY domain of *At*WRKY1 (*Arabidopsis* WRKY1 protein, a member of WRKY family).^[^ ^32^, ^33^ ^]^ We are able to distinguish each distinct dynamic stage of the DBP binding process (Figure 1c), demonstrating the capability of revolutionizing the current techniques used for studying complex biomolecular interaction processes.

## 2 Results and Discussion

The DNA‐binding protein in our experiments, WRKY1N, is the N‐terminal WRKY domain of *Arabidopsis* WRKY1 protein (*At*WRKY1 protein) which performs a good affinity to the W‐box DNA sequence.^[^ ^33^, ^34^ ^]^ Isothermal titration calorimetry (ITC) experiments were conducted to evaluate the binding affinity of the wild‐type WRKY1N and DNA (Figure S2a, F1‐DNA in Table S1, Supporting Information). The binding ratio of WRKY1N and DNA is 1:1, and the dissociation equilibrium constant *K*_D_ is about 0.1 µM. The crystal structure analysis of the WRKY1N–DNA (F1) complex further indicates that the –G6’G7’T8’C9’– sequence of the Crick strand is the main recognition sequence. The Y119 and K122 residues in WRKY1N play a major role in DNA recognition by having a strong interaction with the two G bases (K122 with G6’ and G7’) and the C base (Y119 with C9’) (Figure S3, Supporting Information).^[^ ^34^ ^]^ Further mutations of G7’C9’ in DNA and K122 in WRKY1N greatly reduce the binding affinity of DNA and WRKY1N (Figure S2c,h, Supporting Information). These results demonstrate that WRKY1N has a high binding affinity with the W‐box DNA, and the binding interaction between WRKY1N and F1‐DNA is sensitive to the GGTC sequence in the Crick strand and the key amino (Y119 and K122) in WRKY1N.

To build a single‐molecule monitoring system based on SiNW FETs (Figure 1a), we used a single‐DNA modification protocol reported in our previous works.^[^ ^29^, ^30^ ^]^ In brief, we first treated the primary SiNWs with gas‐phase triethoxy (3‐succinate propyl) silane to obtain surface‐carboxylated SiNWs, which were used in the following processes of FET fabrication and DNA conjugation (Figures S4−S6, Supporting Information). Polymethyl methacrylate (PMMA) was used as the mask to cover the device, and a nanogap was generated with electron‐beam lithography (EBL) to expose certain activated surfaces of SiNWs. After that, a Cy3‐labeled DNA (1 nmol L^−1^ solution) was conjugated to the SiNW surface (Figure S5, the DNA sequence information is in Table S1, Supporting Information). The Cy3 label enabled us to further characterize the device with Stochastic Optical Reconstruction Microscopy (STORM). The single fluorescence spot on the silicon nanowire (Figure 1b and Figure S7, Supporting Information) clearly guarantee a single DNA molecule bonded on the SiNW surface.

**Figure 2:**
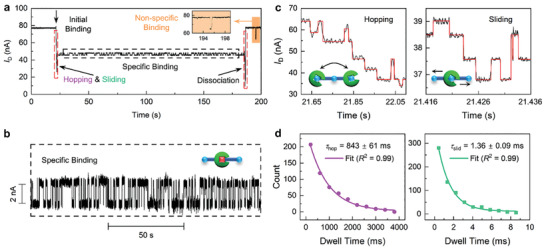
Representative real‐time current trajectories of a whole DNA–WRKY1N interaction process. a) 200 s real‐time trajectory showing the complete interaction process of F1‐DNA and WRKY1N including initial binding, WRKY1N searching for the cognate site (hopping & sliding), specific binding, and dissociation. The specific binding process in the black dashed box is magnified in Figure 2b. The detailed searching process in the left red dashed‐line box of hopping and sliding is magnified in Figure 2c. Top right inset (orange) shows a single‐stage signal representing non‐specific binding and dissociation of WRKY1N. b) Magnified view of the specific binding process. c) Magnified representative real‐time trajectories of the 1D facilitated protein searching process in different patterns, hopping and sliding (black, raw data; red, idealization with a step finding program). d) Dwell time distribution (dots) of the hopping (purple) and sliding (green) process showing a single exponential decay fits (curve lines), generating the average dwell time *τ*_hop_ (843 ± 61 ms, *n* = 550) and *τ*_slid_ (1.36 ± 0.09 ms, *n* = 663).

After device fabrication, to accurately detect the binding process, Conductance (current) time signals have been recorded with a high‐speed sampling rate (28 800 Sa s^−1^) when a constant source‐drain bias voltage was applied. We first tested a single‐DNA modified device in the blank buffer (10 mmol L^−1^ HEPES, 100 mmol L^−1^ NaCl, pH = 7.0) at different temperatures. There was no significant signal switching during the measurement (Figure S9, Supporting Information), which confirms both DNA‐modified devices and DNAs have the good stability at this ambient temperature range. In addition, the bare device has also been measured in the WRKY1N solution as another control experiment. The results consistently show no obvious interactions between the bare device and the WRKY1N protein (Figure S10, Supporting Information). Interestingly, adding WRKY1N protein to the F1‐DNA modified chip induces various current signal responses, which reflect the dynamic process of DNA‐WRKY1N interactions (**Figure** **2**). Salt (NaCl) concentration‐dependent experiments were then carried out to choose the optimal condition at 100 mmol L^−1^ for the subsequent systematical study (Figure S11, Supporting Information). The current signals became faster and more complex as the salt concentration increased and showed less regularity at lower and higher concentrations than at 100 mmol L^−1^ (Figure S11a–c, Supporting Information). No significant signals were observed because of the screening effect when the salt concentration reached 500 mmol L^−1^ (Figure S11d, Supporting Information). The significant current drop in Figure 2a should originate from the initial binding of WRKY1N protein with DNA. The theoretical isoelectric point (pI) of WRKY1N protein is about 9.20, meaning that WRKY1N is positively charged in the buffer (pH = 7.0). In this case, according to the charge transport mechanism of a *p*‐type silicon nanowire, the binding of WRKY1N leads to a current decrease.^[^ ^29^, ^30^, ^35^ ^]^ Single‐pulse signals with a short dwell time (≈s) were observed (Figure 2a, inset), representing a non‐specific binding of WRKY1N onto DNA followed by a direct dissociation without subsequent searching. WRKY1N first interacts with DNA after collision, causing the current decrease and performs an instantaneous non‐specific binding with DNA followed by direct dissociation, which induce the recovery of the conductance. The increase and decrease of the current caused by WRKY1N binding on DNA show a multi‐step process, which was repeatedly observed (Figure 2a–c). The appearance of the multi‐step signals indicates a transient change of charge density near the SiNW surface. This could result from the distance change between the positively charged WRKY1N protein and the SiNW, enabling further analysis of the intermediate steps—the 1D searching process of WRKY1N along the DNA chain. Single‐base resolution can be achieved by the high temporal resolution of the electrical detection as discussed below (the sampling interval of ≈34 µs using here).

The 1D searching process of WRKY1N mainly resulted in two different signal behaviors (Figure 2c). In the slow interaction pattern, longer dwell times and larger current changes (Δ*I*, ≈10 nA) represent a more dramatic interaction corresponding to the WRKY1N hopping across the DNA chain. In this process, the protein does not totally dissociate from DNA into the solution, but leaves the ion radius of DNA and rebinds to DNA after a large translocation between DNA fragments,^[^ ^11^, ^13^ ^]^ leading to a larger current change (Figure 2c left and Figure S12, Supporting Information). In comparison, much more short dwell times and small Δ*I* (≈1 nA) occur in the fast interaction pattern, corresponding to a minor motion amplitude and a higher frequency (Figure 2c right and Figure S13, Supporting Information). According to an all‐atom molecular‐dynamics (MD) simulation for diffusion dynamics based on the crystal structure of WRKY1N–DNA complex,^[^ ^36^ ^]^ this high‐frequency process (µs to ms scale) is consistent with the 1D sliding process, where WRKY1N translocates along the major groove of the DNA chain in a loose binding state. The current steps of the sliding process usually occurred with a uniform step height and the steps changed with the minimum height or its integer times (Figure 2c right and Figure S13, Supporting Information), which indicates a basic stepping unit for single‐base‐pair movements. It is worth mentioning that real‐time monitoring captured the reversible step‐like signals, which indicates that WRKY1N can search back‐and‐forth along DNA to locate the specific binding site (Figure 2c and Figures S12 and S13, Supporting Information) through random hopping or sliding. To quantify the kinetics properties, we used a step‐fitting algorithm^[^ ^37^ ^]^ to evaluate the two different multi‐step processes (Figure 2c). The distributions of the dwell time can be well fitted to a single‐exponential decay function, generating the average dwell times (*τ*_hop_ = 843 ± 61 ms and *τ*_slid_ = 1.36 ± 0.09 ms, Figure 2d). According to the analysis, the magnitude of the 1D diffusion coefficient (sliding) scale (10^5^–10^6^ bp^2^ s^−1^) is consistent with other results from previous experimental or theoretical works,^[^ ^10^, ^15^, ^16^, ^20^, ^25^, ^26^, ^38^ ^]^ where the single‐base‐pair searching process was evaluated. The single‐base‐pair stepping cycle of WRKY1N along the major groove of DNA was also captured in the MD simulation as hydrogen bonds break and reform at the protein–DNA binding interface.^[^ ^36^ ^]^ This corroborates that DBP–DNA searching with single‐base‐pair resolution in a whole monitoring process has been successfully observed on our single‐molecule electrical detection platform.

Along with the multi‐step signals during real‐time monitoring, there were long‐term uniform two‐level current fluctuation signals (mixed inside) with relatively small amplitude (1–2 nA, Figures 2a,b). According to the chronological order of the different signal patterns and the characteristics of the current changes, this two‐level oscillation signals is very likely originated from the periodic interaction between WRKY1N and the specific DNA binding site. To further decode the specific binding behavior of the DBP–DNA interaction, a series of specially designed different DNA sequences with WRKY1N have been carried out for long‐time measurements. For non‐specific sequences (F2 and F3 in Table S1, Supporting Information), only large current drops and multi‐step signals were observed (Figure S14a, Supporting Information). This refers to the non‐specific binding of DNA with WRKY1N and the 1D search process of WRKY1N along the DNA chain. It also verifies the uniform periodic two‐level signal only appears with the coexistence of DNA and specific binding sequence (GGTC), thus confirming our speculation that the bistable oscillation signal results from the specific binding.

To systematically study the DNA length effect on the DBP–DNA dynamic interaction, we tested three single‐site DNAs (F1, F4, and F5 in Table S1, Supporting Information) with different lengths (Figure S14b, Supporting Information). All three specific DNAs caused the long‐term periodic oscillation and the signal intensity was positively correlated with the DNA length. The interaction signal between WRKY1N and short DNA (F1 and F4) is more regular and homogeneous in comparison with the signal of long DNA (F5) where a small amount of the third‐state burst signal starts to appear, indicating more complex interaction patterns in the longer DNA. To simplify the system, we mainly used the 16 bp length DNA (F1) in the subsequent experiments to define the dependence of protein concentration and operation temperature.

In the WRKY1N concentration‐dependent experiments, the current distribution and frequency of the bistable signals show no significant differences at different WRKY1N concentrations (Figures S15 and S16, and Table S2, Supporting Information), confirming that the regular periodic oscillation signal originates from a single WRKY1N–DNA complex (fixed on the SiNW surface) rather than an on/off behavior between DNA and WRKY1N or multiple binding of another WRKY1N. For more rigorous considerations, control experiments with single‐WRKY1N modified devices (Figure S8, Supporting Information) were also performed to evaluate signal fluctuations caused by conformational changes of WRKY1N. During the measurements of WRKY1N devices (Figure S17, Supporting Information) in an empty buffer, only slight fluctuations appeared, which shows that WRKY1N only has a negligible structural perturbation (Figure S17 left, Supporting Information). After the addition of F1‐DNA (10 µmol L^−1^), step‐like signals subsequently with periodic current oscillation occurred. The much larger oscillation amplitude is from the WRKY1N binding with DNA (Figure S17 right, Supporting Information) as the binding of DNA, which is negatively charged, could induce more carriers inside the *p*‐type SiNW channel and improve the conductivity.

**Figure 3:**
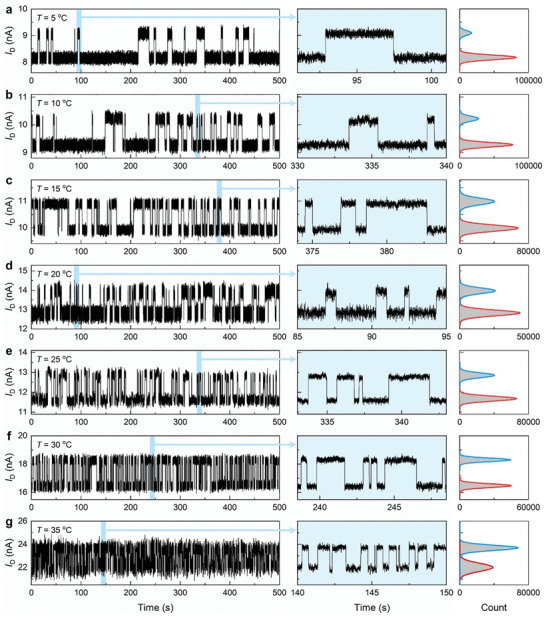
Real‐time trajectories of the DBP–DNA specific binding interaction at different temperatures. a–g) 500 s real‐time data sets on the left panels recorded under seven different temperatures in a fixed WRKY1N concentration solution (100 mmol L^−1^ NaCl, 10 mmol L^−1^ HEPES, and 10 µmol L^−1^ WRKY1N), from a) 5 °C, b) 10 °C, c) 15 °C, d) 20 °C, e) 25 °C, f) 30 °C to g) 35 °C. The middle panels (marked with blue) show the 10 s magnified view of each trace and the right panels are the corresponding current histograms of each trace.

For a further investigation of this DNA‐WRKY1N complex, temperature‐dependent experiments have been performed with a DNA‐modified device. **Figure** **3** shows 500‐second real‐time current trajectories at seven different temperatures (5–35 °C) recorded from the same device at a fixed WRKY1N concentration (10 µmol L^−1^ in a 10 mmol L^−1^ HEPES buffer). Typical two‐state periodic oscillation signals of this binding complex have been recorded at each temperature (Figure 3 left). The statistical histograms show a changing distribution of the high/low current states (Figure 3 right). The increasing temperature shortened the dwell times of both states and accelerated the motion of the complex. And the high state gradually became predominant when the temperature reached 35 °C (Figure 3 and Figure S18h,i, Supporting Information). What is left unclear, however, is how the current change by K122 residue swing is bigger than that by diffusion, whether it originates from the long‐range conformation change induced by K122 residue swing, the compact structure formed at the binding site within the Debye length or another associative mechanism.

**Figure 4:**
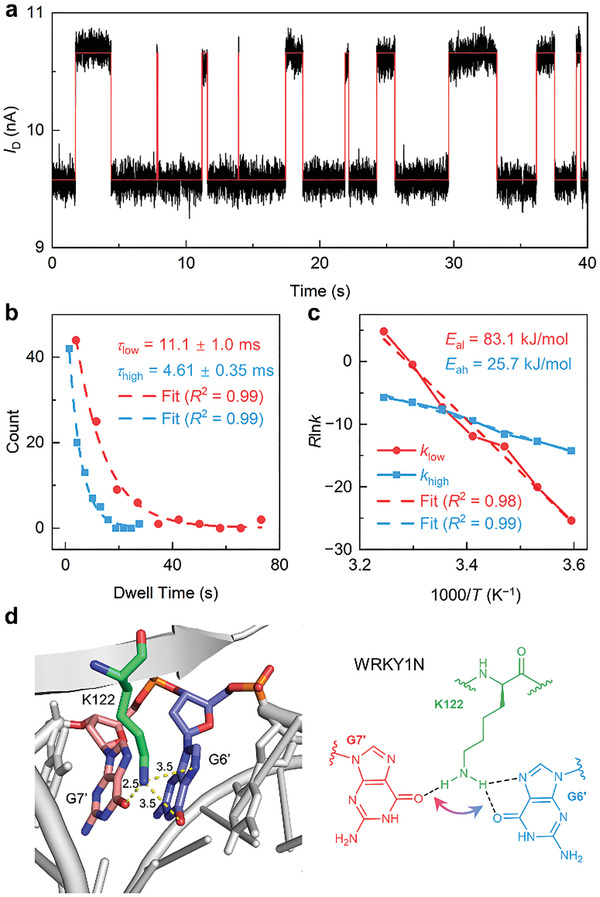
Kinetic properties of the DNA–WRKYN complex interaction process. a) Typical real‐time data (black line) of single DNA interaction with WRKY1N (10 µmol L^−1^) at 20 °C and idealized data fitted by a QuB software. b) The distribution of the dwell times for low (red dot) and high (blue square) current states at 10 °C. Dashed lines represent the single‐exponential fittings (red for the low state and blue for the high state), which generate the average dwell times *τ*_high_ (11.1 ± 1.0 ms, *n* = 90) and *τ*_low_ (4.61 ± 0.35 ms, *n* = 90). The results of other temperature are shown in Figure S18 (Supporting Information). c) Arrhenius plots of the signal changes between two different current states (red for the low state and blue for the high state) of the specific binding process. d) Left panel shows the crystal structure of K122 with G6’ and G7’ in WRKY1N–DNA complex (the structure was adopted from ref. [34], PDB code: 6J4E). Right panel shows the schematic of the dynamic process. The double arrow shows the reversible swinging of K122 and the dynamic enhancement of different hydrogen bonds with different G bases.

The QuB software, which is widely applied to analyze the single‐molecule process, was used to idealize the oscillation signal (**Figure** **4**a) and extract the dwell times of different current states based on the hidden Markov model.^[^ ^39^ ^]^ Figure 4b shows the dwell‐time distributions of both current states in 10 µmol L^−1^ WRKY1N solution at 20 °C. Both distributions can be well fitted to a single‐exponential decay function. As a result, the average dwell times of the high state (*τ*_high_) and the low state (*τ*_low_) at different temperatures can be extracted (Figure S18a–f, Supporting Information). The transformation rates are derived from the dwell times, *k*_high_ = 1/*τ*_high_ and *k*_low_ = 1/*τ*_low_, further generating the Arrhenius plots (Figure 4c). The Arrhenius plots of *k*_high_ and *k*_low_ can be well linearly fitted, showing a good Arrhenius behavior for the forward and reverse transformation. The activation energies can be figured out from the fitting equations (for high state to low state, *E*_ah_ = 25.7 ± 1.2 kJ mol^−1^ and for low state to high state *E*_al_ = 83.4 ± 4.7 kJ mol^−1^). This high energy barrier results in a relatively slow conversion between these two states. The higher activation energy of the low state, *E*_al_, indicates the lower state is dominant in most of the temperature‐dependent experiments. The difference in the activation energies is about 55.7 kJ mol^−1^ (≈13.3 kcal mol^−1^), which is consistent with the energy level of hydrogen bonds in the protein–DNA interaction.^[^ ^40^, ^41^, ^42^ ^]^ The experiments with different protein concentrations show similar results (Figure S19, Supporting Information).

According to the crystal structure of the WRKY1N–DNA complex, K122 forms a pair of hydrogen bonds with G6’ and G7’ bases, further stabilizing the whole structure. To find out the contribution of K122 to the oscillation signals, we mutated K122 to A122 and then tested the F1‐DNA modified device in the mutant type WRKY1N (MT‐WRKY1N) solution. After K122A mutation, less regular oscillation signals with much higher frequency and shorter duration time were recorded (Figure S20, Supporting Information). This result indicates that the stability of the MT‐WRKY1N–DNA complex is much weaker than the wild‐type complex, consistent with the ITC result (Figure S2, Supporting Information), and also proved the connection between the existence of K122 and the bistable oscillation signal of the binding complex.

Based on the electrical signals in the whole interaction process, the long‐last (up to tens of minutes) regular bistable oscillation signals suggest that such a binding complex is a sustainable, dynamic system of which the crystal structure vision can only provide us with a static cross‐section. These two transient states of the stable complex with such tiny differences often tend to be masked in ensemble experiments. However, they can be revealed by single‐molecule techniques. In combination with the crystal structure of the WRKY1N–DNA complex, the oscillation signals may give a finer correlation information between the structure and the dynamic behavior. From the high‐resolution crystal structure (Figure S3a, Supporting Information and Figure 4d), K122 and two G bases (G6’, G7’) in the WRKY1N–DNA complex form a pair of asymmetric hydrogen bonds. Due to both lattice restriction and crystal contact, the complex only performs an average static uniform conformation in the crystal. However, in the solution, the protein–DNA complex with more degrees of freedom can perform a long‐range vibration,^[^ ^43^, ^44^, ^45^ ^]^ inducing a collective motion between the two weakened conformations with the alternative enhancement of K122‐G6’ or K122‐G7’. Since K122 is indispensable to the specific‐binding conformation, the swinging of K122 between two bases is bound to disturb the whole conformation of the complex, inducing two degenerated states in the electrical monitoring. However, the intricate interplay of other residues and DNA in this stable combination prevents WRKY1N from dissociation and keeps it closely attached to the cognate site, which accounts for the high‐energy barrier and the long average dwell times of the two‐conformation switching. The conformation with K122 closer to G7’, as in the crystal structure, may exhibit more stability and become predominant at a lower temperature. With increasing the temperature, the energy barrier becomes easier to overcome, and the distribution and transformation rate of two metastable states become similar (Figure S18h,i, Supporting Information). This collective motion of the single protein–DNA complex suggests biomolecules performs persistent vibration even when they are closely binding with their partner molecules, which implies that life always lies in movement even at the single‐molecule level.

## 3 Conclusion

To summarize, we demonstrate an unprecedented direct, high‐performance silicon nanowire FET nanocircuit with high temporal resolution and high sensitivity to monitor the single‐molecule binding process of the WRKY1N protein with W‐box DNA in real time. By measuring the current signal of the whole interaction process of the single DNA with protein, for the first time the chronological complete process of DBP searching for the DNA binding site with in situ single‐base‐pair resolution has been observed, which includes three different stages: 3D diffusion of protein onto DNA through non‐specific binding, 1D searching along DNA chain, and finally specific binding to the cognate site. Additionally, the long‐last collective motion of the WRKY1N–DNA complex is highly correlated to the dynamic swinging of K122‐G6’ and K122‐G7’. We believe that this ultrasensitive detection platform is ready to be applied to a variety of label‐free chemo‐detections/bio‐detections at the single‐molecule or single‐event level, holding great promise for the development of low‐noise multiplex detection electronics for accurate molecular and even point‐of‐care clinical diagnosis in combination with current microelectronics.

## 4 Experimental Section

### Protein Expression and Purification

The codon‐optimized N‐terminal DNA binding domain (DBD) of the Arabidopsis WRKY1 was constructed into the pET21b vector with C‐terminal his‐tag and subsequently transformed into the E. coli strain BL21 (DE3). Protein overexpression was induced by the addition of isopropyl *β*‐D‐1‐thiogalacto‐pyranoside to a final concentration of 0.5 × 10^−3^ m. Cells were left to grow overnight at 18 °C. Bacteria were then collected and resuspended in buffer A (25 × 10^−3^ m HEPES, pH 7.0, 1.0 M NaCl) before sonication and centrifugation. Subsequently, the supernatant was loaded onto a Ni‐chelating column (GE Healthcare, USA), and target protein was eluted by imidazole at a concentration of 200–500 × 10^−3^ m, followed by size‐exclusive chromatography (Superdex 75, GE Healthcare) for final purification in buffer C (25 × 10^−3^ m HEPES, pH 7.0, 100 × 10^−3^ m NaCl). Purified protein was concentrated and stored at −80 °C after flash freezing by liquid nitrogen.

### Isothermal Titration Calorimetry (ITC) Assays

The DNA samples for ITC were purchased from ThermoFisher (Thermo Fisher Scientific, USA) and dissolved in buffer C (25 × 10^−3^ m HEPES, pH 7.0, 100 × 10^−3^ m NaCl). The DNA samples were heated to 95 °C, slowly cooled down to anneal to room temperature, and then further purified by size‐exclusive chromatography (Superdex 75, GE Healthcare) to remove single‐stranded DNAs. The purified WRKY1N and DNA were kept in buffer C (25 × 10^−3^ m HEPES, pH 7.0, 100 × 10^−3^ m NaCl) before titration. To determine the affinity between WRKY1N and DNA, 0.25 × 10^−3^ m WRKY1N was titrated into 0.026 × 10^−3^ m DNA using an ITC200 (GE Healthcare) at 20 °C. The thermograms were integrated by Origin software and fitted in the “one set of sites” mode.

### Device Fabrication

The nanowire growth procedure is similar to those reported in the previous studies.^[^ ^46^, ^47^ ^]^ Gold nanoparticles (AuNPs, Sigma‐Aldrich, the average diameter of ≈20 nm) were used as catalysts dispersing on silicon wafers with a 300 nm thick thermal oxide layer. Boron‐doped *p*‐type SiNWs were synthesized at 470 °C for about 20 min by using 2.5 sccm Si_2_H_6_ (Matheson Gas Products, 99.998% Purity) as reactant gas, 0.25 sccm B_2_H_6_ (100 ppm, diluted in H_2_) as a *p*‐type dopant, and 7.0 sccm H_2_ as the carrier gas. The wafer on which silicon nanowires and 10–20 µL triethoxy (3‐succinate propyl) silane (TESPSA, 95%, J&K) were grown was placed in a sealed container which was then heated at 120 °C for 2 h. After the vapor modification, modified SiNWs were transferred to a 1.4 cm × 1.8 cm silicon substrate with a 1000 nm thick thermal oxide layer. The electrode patterns were defined by a standard UV lithography (BG‐401A, China electronics technology Group Corporation). After the etching of SiNWs with a buffered HF solution (40% NH_4_F:40% HF, 7:1) to remove the oxide shell, 8 nm Cr and 80 nm Au were deposited through thermal evaporation (ZHD‐300, Beijing Technol Science) to form metal electrodes. A 30 nm thick SiO_2_ protective layer was then deposited through electron beam thermal evaporation (TEMD‐600, Beijing Technol Science) in order to passivate the contact interface. After lift‐off with acetone, the surface‐modified SiNW FET devices were obtained.

### Electrical Characterization and Single DNA Decoration

The characterization of the SiNW FET devices was carried out by using an Agilent 4155C semiconductor analyzer and a Karl Süss (PM5) manual probe station.^[^ ^29^ ^]^ In the present strategy of device modification, since DNA had better structural stability and environmental tolerance than proteins, a single DNA with a specific binding sequence on the surface of SiNWs was immobilized. The DNA binding protein (WRKY1N) was added during the monitoring of the system. The disturbances on the structure and function of the protein caused by the immobilization can be avoided as a result. Polymethyl methacrylate (PMMA) was used as the mask to cover the device, and a nanogap was generated with EBL to expose certain activated surfaces of SiNWs.^[^ ^29^, ^47^ ^]^ The devices were then treated with the Cys3‐labeled amino‐terminal DNA (1 nmol L^−1^). The amino reacts with TESPSA terminal carboxylic acid on the surface of silicon nanowires, and the DNA molecules was finally immobilized on the surface of SiNWs. The Cy3‐labeled DNA was further characterized by the Stochastic Optical Reconstruction Microscopy (STORM) under the excitation light of 561 nm wavelength and only single point fluorescent can be observed on the SiNW (Figure 1b and Figure S6, Supporting Information). The DNA sequences used in this work are listed in Table S1 (Supporting Information).

### Real‐Time Electrical Measurements

The silicon substrate with single DNA decorated devices was covered by a PDMS cube with a hole of ≈2 mm diameter as a reaction chamber. A 50 µL WRKY1N solution with a specific concentration was then dropped into the microchamber. The INSTEC hot/cold chuck with a proportion‐integration‐differentiation control system (± 0.001 °C), and a liquid nitrogen cooling system was used to precisely control and maintain the testing temperature. The source‐drain and gate biases were set at DC 300 and 0 mV, respectively, by an HF2LI Lock‐in Amplifier (Zurich Instruments) in all the real‐time electrical measurements. The source–drain current through selected SiNW device was amplified by a DL1211 preamplifier operating at 10^7^ V A^−1^ gain and collected by the HF2LI Lock‐in Amplifier with a bandwidth of 5 kHz low‐pass filter at sampling rates of 28.8 or 7.2 kHz.

### Statistical Analysis

The current data were recorded with a combination of HF2LI Lock‐in Amplifier (Zurich Instruments) and DL1211 preamplifier at a sampling rate of 28.8 or 7.2 kHz. The raw data with high bandwidth (10 kHz) were then used to reduce the signal noise of the circuit by a low‐pass Butterworth filter at a frequency of 2 kHz. The additional filtered processes were carried out by MATLAB 2016b. QuB or a step finding algorithm was then used to idealize the filtered data based on the hidden Markov model.^[^ ^37^, ^39^ ^]^ The dwell time of each signal event and the number of total events were extracted after the idealization. The extracted data were then analyzed with Origin 9.0. The dwell time was then fitted to a single‐exponential decay function and the average dwell time was generated. Data are presented as mean ± SD. For statistical test, One‐way ANOVA testing followed by a Tukey post‐hoc test was carried out across groups. Significance was defined as *p* ≤ 0.05. Statistical analysis was carried out using Origin 9.0.

## Conflict of Interest

The authors declare no conflict of interest.

## Supporting information

Supporting Information

## Data Availability

The data that support the findings of this study are available from the corresponding author upon reasonable request.
