# Supplementary material for: Complete Mapping of DNA‐Protein Interactions at the Single‐Molecule Level
Source: Adv Sci (Weinh). 2021 Oct 5;8(23):2101383. doi: 10.1002/advs.202101383 (PMC8655176; doi:10.1002/advs.202101383)
Supplement: Supplementary file 1 — Supporting Information [file ADVS-8-2101383-s001.pdf]

© 2021 Wiley-VCH GmbH

Supporting Information

for *Adv. Sci.,* DOI: 10.1002/advs.202101383

Complete Mapping of DNA-Protein Interactions at the Single-Molecule Level

Wenzhe Liu, Jie Li, Yongping Xu, Dongbao Yin, Xin Zhu, Huanyan Fu, Xiaodong Su, and Xuefeng Guo**

Supporting Information

Complete Mapping of DNA-Protein Interactions at the Single-Molecule Level

Wenzhe Liu, Jie Li, Yongping Xu, Dongbao Yin, Xin Zhu, Huanyan Fu, Xiaodong Su, and Xuefeng Guo**

W. Liu, Dr. J. Li, D. Yin, Prof. X. Guo
State Key Laboratory for Structural Chemistry of Unstable and Stable Species
Beijing National Laboratory for Molecular Sciences
National Biomedical Imaging Center
College of Chemistry and Molecular Engineering, Peking University
Beijing 100871, P. R. China
E-mail: guoxf@pku.edu.cn

Dr. J. Li
Shenzhen Bay Laboratory
Shenzhen 518132, P. R. China

Dr. Y. Xu, Prof. X. Su
State Key Laboratory of Protein and Plant Gene Research
Biomedical Pioneering Innovation Center (BIOPIC), Peking University
Beijing 100871, P. R. China
E-mail: xdsu@pku.edu.cn

X. Zhu, H. Fu, Prof. X. Guo
Center of Single-Molecule Sciences
Frontiers Science Center for New Organic Matter
Institute of Modern Optics
College of Electronic Information and Optical Engineering
Nankai University
38 Tongyan Road, Jinnan District, Tianjin 300350, P. R. China.

Table of Contents

S1. Protein purification and characterization

Figure S1. Strategical demonstration of single-molecule experiments of the DNA-DBP system

Figure S2. ITC results of the WRKY1N-DNA binding system

Figure S3. Crystal structure analysis of a WRKY1N-DNA complex

S2. Device fabrication and characterization

Figure S4. Verification of DNA modification

Figure S5. Schematic of the single-DNA modified device fabrication procedure

Figure S6. Optical and SEM images of a SiNW FET array.

Figure S7. Characterization of single-DNA-modified SiNW FET devices.

Figure S8. AFM image of a single WRK1N modified SiNW-FET device.

S3. Real-time current measurement and dynamic analysis

Figure S9. Controlled experiments of a DNA-modified device in the blank buffer

Figure S10. Controlled experiments of bare SiNW devices in the WRKY1N solution

Figure S11. Measurements of salt concentration-dependent experiments.

Figure S12. Slow multi-step signals of different DNA-modified devices

Figure S13. Fast multi-step signals of different devices

Figure S14. Measurements of WRKY1N protein interaction with different DNAs

Figure S15. Measurements of WRKY1N concentration-dependent experiments

Figure S16. Comparison of signal properties in different WRKY1N concentration

Figure S17. Temperature-dependent experiments of the WRKY1N-modified device

Figure S18. Analysis of temperature-dependent experiments (10 μmol L^{-1} WRKY1N)

Figure S19. Analysis of temperature-dependent experiments (100 μmol L^{-1} WRKY1N)

Figure S20. Experiments of mutant WRKY1N (MT-WRKY1N)

Table S1. DNA sequence information

Table S2. Kinetic and thermodynamic analysis of concentration-dependent experiments

S1. Protein purification and characterization

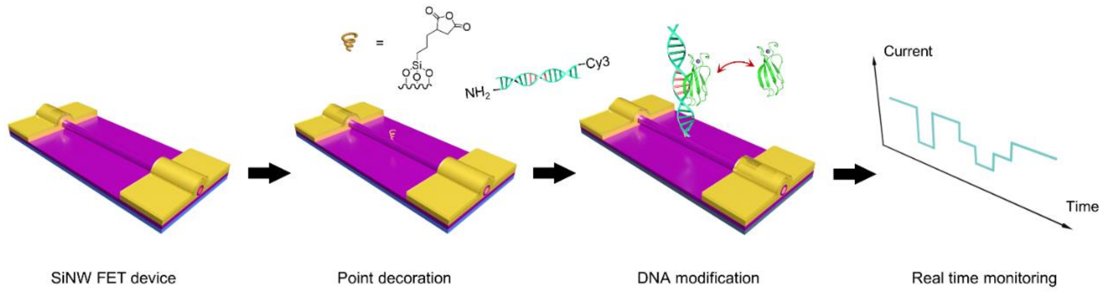

Figure S1. Strategical demonstration of single-molecule experiments of the DNA-DBP system. A single-molecule DNA biosensor is constructed on silicon nanowire field-effect transistor (FET) devices and the electrical signals are measured and recorded so as to realize real-time monitoring of the protein-DNA binding process with high time resolution.

Details of protein expression/purification and Isothermal Titration Calorimetry (ITC) Assays are provided in the Experimental Section of the main text. The ITC results[1] (Figure S2, adopted from Ref. 1) showed that the binding ability of DNA to WRKY1N decreased by 1–2 orders of magnitude due to the mutation of the recognition sequence (GGTC). The relative position of GGTC in DNA had little effect on the binding ability. K122 mutation of WRKY1N also led to a significant decrease in the binding ability.

WRKY1N sequence:

MVHTQTLFDIVNDGYRWRKYGQKSVKGSPYPRSYYRCSSPGCPVKKHVERSSHDTK
LLITTYEGKHDHDMPPLEHHHHHH.

Number of amino acids: 80.

Molecular weight: 9476.64 D.

Theoretical pI: 9.20.

All structure figures were generated by PyMOL. According to Figure S3, GGTC is the core sequence recognized by WRKY1N protein. Y119 and K122 residues interact directly with G and C in the sequence.

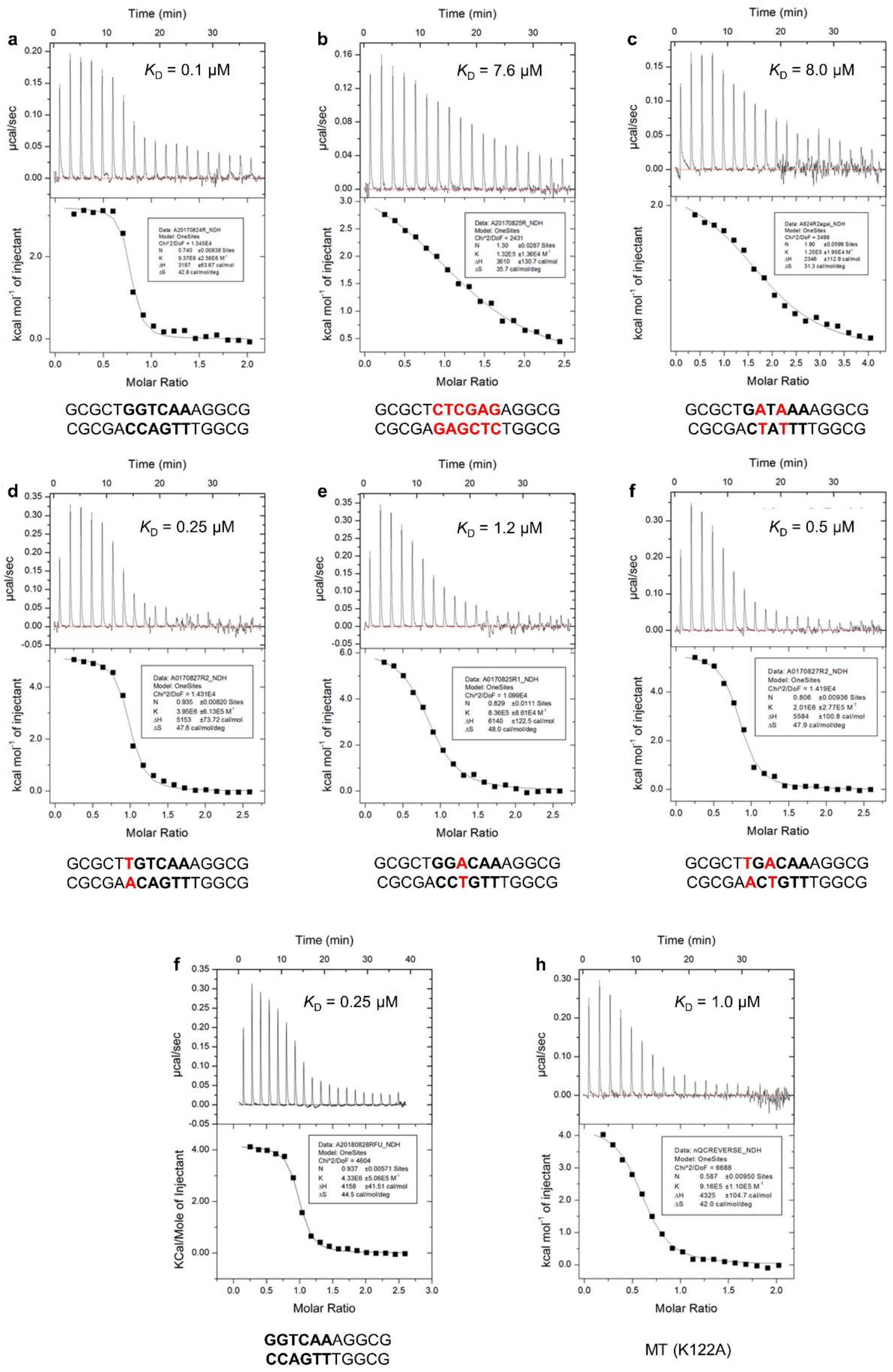

Figure S2. ITC results of the WRKY1N-DNA binding system[1]. The DNA sequences are shown under each graph. a) The ITC result of wild-type WRKY1N (WT-WRKY1N) and wild-type DNAs (WT-DNA, 16 nt), K_D = 0.1 μmol L^{-1}. The cognate sequence is shown in bold. b) The ITC result of WT-WRKY1N and non-specific DNAs (16 nt), KD = 7.6 μM. c–f) The ITC results of WT-WRKY1N and mutant-type DNAs (MT-DNA, 16 nt). The mutant bases are shown in red in each panel. g) The ITC result of WT-WRKY1N and asymmetric cognate DNAs (11 nt). h) The ITC result of mutant-type WRKY1N (MT-WRKY1N) and WT-DNAs (16 nt).

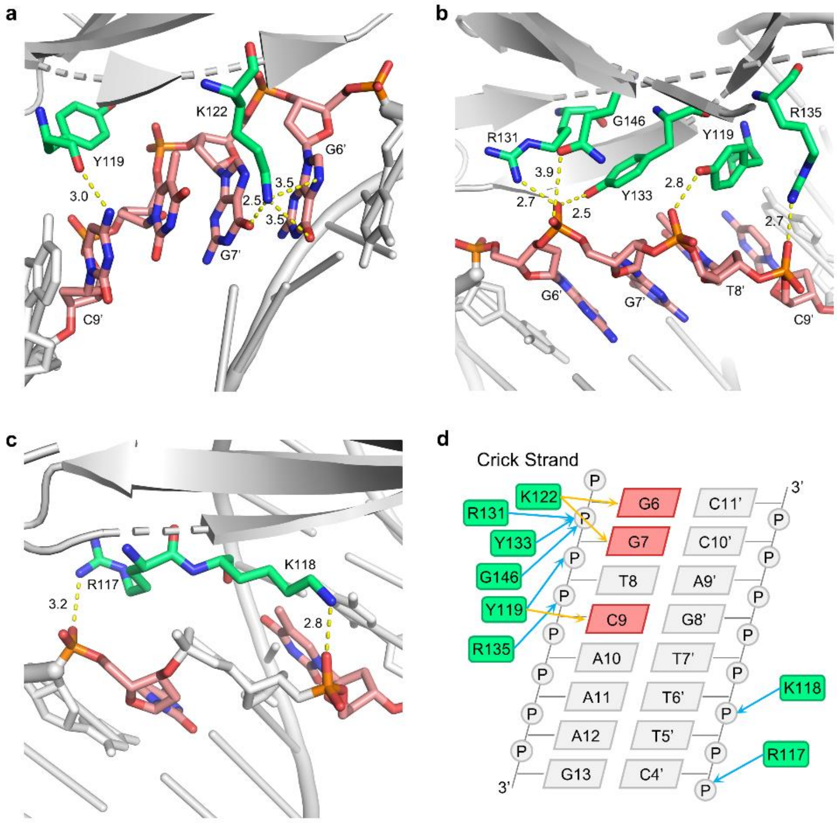

Figure S3. Crystal structure analysis of a WRKY1N-DNA complex[1]. a–c) The crystal structure of a WRKY1N-DNA complex (adopted from Ref. 34, PDB code: 6J4E) at different angles shows the hydrogen bonds between protein residues and DNA (a, specific bases; b and c, phosphate skeleton). d) Schematic diagram of the interaction between protein residues (green) and DNA. Yellow arrows represent hydrogen bonds between residues and bases. Blue arrows represent hydrogen bonds between residues and phosphate skeleton. Protein residues and GGTC sequence have the strong interaction.

S2. Device fabrication and characterization

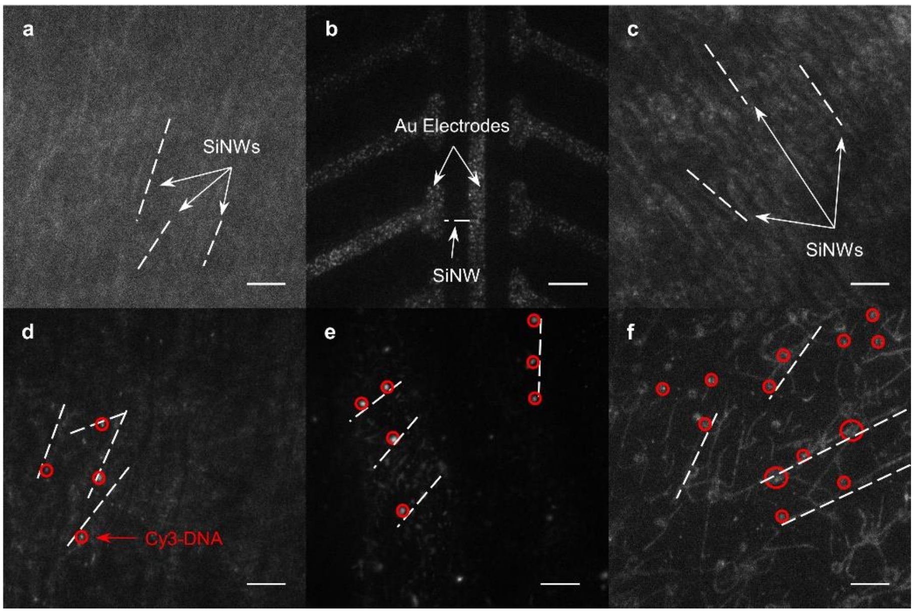

Figure S4. Verification of DNA modification with florescent pictures. a) Silicon nanowires without any treatment. b) SiNWs FET devices only treated with 100 μmol L^{-1} Cy3-DNA. There is no adsorption of DNA on the surface of the device. c, d) (3-Glycidoxypropyl) methyldiethoxysilane (GPTMS) modified SiNWs treated by 10 nmol L^{-1} (c) and 100 μmol L^{-1} (d) Cy3-DNA. e, f) Triethoxysilylpropyl succinic anhydride (TESPSA) modified SiNWs treated by 10 nmol L^{-1} (e) and 100μmol L^{-1} (f) Cy3-DNA. The efficiency of DNA modification is much higher. Scale bar: 5 μm. Dash lines denote SiNWs and red circles denote Cy3-DNA.

To further construct the single-molecule DNA + DBP interaction system, we need to ensure that only a single DNA is immobilized. The Cy3-labeled DNA molecules provide the possibility of fluorescence characterization. We first pictured the background of the silicon nanowire device under the fluorescence microscope. The device itself did not show the obvious fluorescence signal, and the Cy3 labelled DNA did not interact with the bare SiNW device. GPTMS-modified SiNWs showed poor conjugation efficiency in comparison with TESPSA, even with a concentration of 100 μmol L^{-1}. However, SiNWs still showed fluorescent signals after TESPA modified SiNWs treated with a 10 nmol L^{-1} DNA solution. In addition, a better distribution of Cy3-DNA was obtained when we treated vapor functionalized silicon nanowires with a 10 nmol L^{-1} DNA solution. To obtain single DNA modified silicon nanowire devices, the modified devices were treated with a 1 nmol L^{-1} DNA solution and the single point fluorescent silicon nanowire devices can be obtained with high probability.

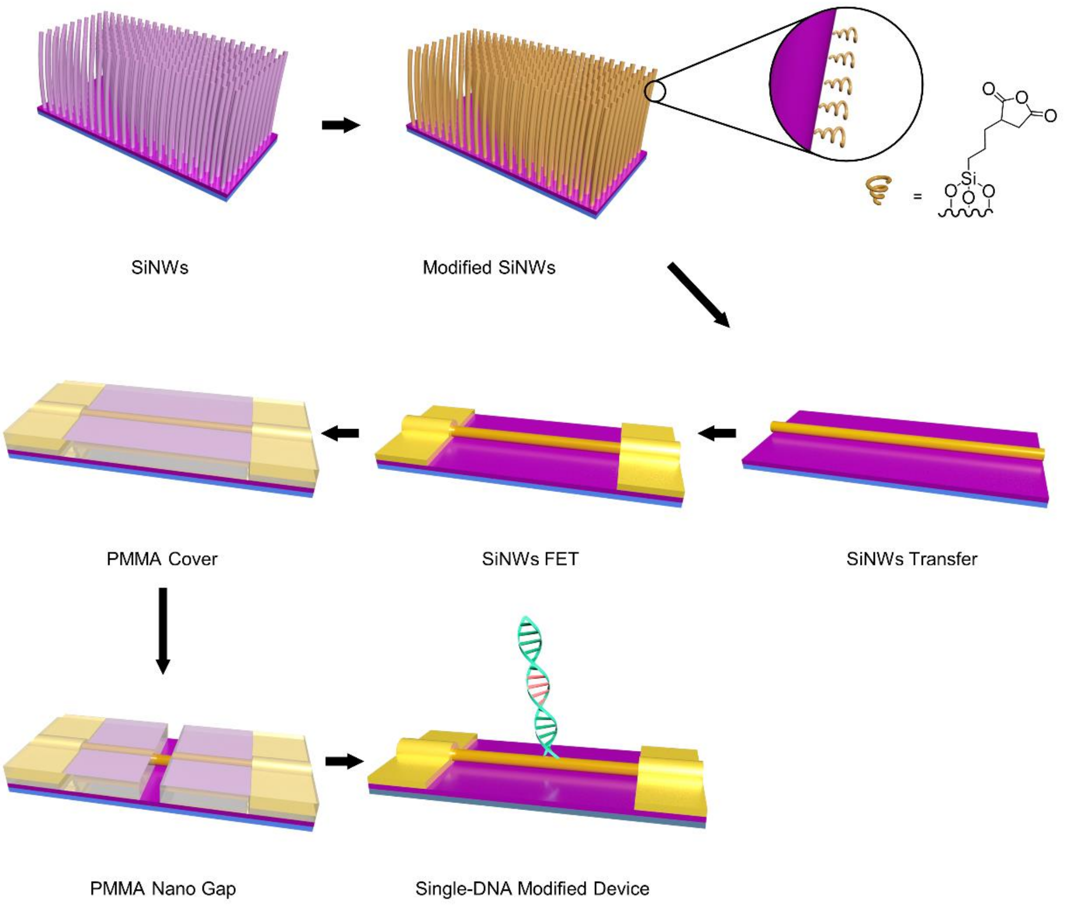

Figure S5. Schematic of the single-DNA modified device fabrication procedure. Detailed description of each step is shown below.

SiNW growth Procedure: The nanowire growth procedure is similar to those reported in the previous studies[2, 3]. Gold nanoparticles (AuNPs, Sigma-Aldrich, the average diameter of ~20 nm) were used as catalysts dispersing on silicon wafers with a 300 nm thick thermal oxide layer. Boron-doped p-type SiNWs were synthesized at 470 ^{o}C for about 20 min by using 2.5 sccm Si_2H_6 (Matheson Gas Products, 99.998% Purity) as reactant gas, 0.25 sccm B_2H_6 (100 ppm, diluted in H_2) as a p-type dopant (B/Si ratio of 1/100000), and 7.0 sccm H_2 as the carrier gas.

Surface modification of SiNWs: We carried out an *in-situ* modification of SiNWs. The wafer on which we grew the silicon nanowires and 10–20 μL triethoxy (3-succinate propyl) silane (TESPSA, 95%, J&K, in a 1.5 mL microtube) were placed in a vacuum desiccator, which was then heated at 120 ^{o}C for 2 hours. This operation also remains the original growth morphology of SiNWs and enables the further transfer of functionalized SiNWs[4, 5].

SiNW transfer and FET fabrication: After vapor modification, the functionalized SiNWs were transferred to a 1.4 cm × 1.8 cm silicon substrate with a 1000 nm thick thermal oxide layer

and well-aligned by mechano-sliding[5, 6]. The electrode patterns were defined by a standard UV lithography (BG-401A, China electronics technology Group Corporation). After the etching of SiNWs with a buffered HF solution (40% NH_4F:40% HF, 7:1) to remove the oxide shell, 8 nm Cr and 80 nm Au were deposited through thermal evaporation (ZHD-300, Beijing Technol Science) to form metal electrodes. A 30 nm-thick SiO_2 protective layer was then deposited through electron beam thermal evaporation (TEMD-600, Beijing Technol Science) in order to passivate the contact interface. After lift-off with acetone, the surface-modified SiNW FET devices were obtained (Figure S6).

Single-molecule DNA decoration: A gap-opening procedure was carried out to decorate the SiNW-FET device with a single DNA molecule. After device fabrication, a PMMA layer (950, A4) was spin-coated (4000 rpm, 45 s) on the surface and then baked at 180 °C for 2 min. The high-resolution electron beam lithography (EBL) was then applied to introduce a design line pattern with a ~5 nm-wide at the specific position to obtain the window precursor (Figure S5). The resist was developed in a mixture of water/isopropanol ($V : V = 1 : 3$) for the lift-off at 4 °C for 1 min with the aid of sonication. After development, the devices were washed by deionized water and dried with a stream of N_2 gas. The modified surface of the SiNW was then exposed with a nanoscale gap for further decoration. The devices were then treated with the Cy3-labeled amino-terminal DNA (1 nmol L^{-1}). The amino reacts with TESPSA terminal carboxylic acid on the surface of silicon nanowires, and the DNA molecules was finally immobilized on the surface of SiNWs. The Cy3-labeled DNA was further characterized by the Stochastic Optical Reconstruction Microscopy (STORM) under the excitation light of 561 nm wavelength and only a single fluorescent point can be observed on the SiNW (Figure 1b and Figure S7).

Single-molecule protein decoration: The procedure is similar to those reported in previous studies[3, 7]. A gap-opening procedure was carried out to decorate the SiNW-FET device with single DNA molecule. After the device fabrication, a PMMA layer (950, A4) was spin-coated (4000 rpm, 45 s) on the surface and then baked at 180 °C for 2 min. The high-resolution electron beam lithography (EBL) was then applied to introduce a design line pattern with a ~5 nm-wide at the specific position to obtain the window precursor (Figure S5). The resist was developed in a mixture of water/isopropanol ($V : V = 1 : 3$) for the lift-off at 4 °C for 1 min with the aid of sonication. After the development, the devices were washed by deionized water and dried with a stream of N_2 gas. The modified surface of the SiNW was then exposed with a nanoscale gap for further decoration. The wafers were then immersed in an aqueous mixed solution of N-hydroxy succinimide (NHS) (20 mM) and 1-ethyl-3-(3-dimethylaminopropyl) carbodiimide (EDC) (10 mM) and allowed to react at room temperature for 1 h (pH = 6.5). The wafers were

washed thoroughly with deionized water and then dried under a stream of N_2 gas. Then the wafers were immersed in the N, N-Dimethylformamide (DMF, J&K, 99.8%, Super Dry, with molecular sieves, J&K Seal) solution of 10 mmol L^{-1} 2-Maleimidoethylamine hydrochloride (2-Maleimidoethylamine HCl, TCI, 93%) for 2 h. After the maleimide modification, the wafers were washed thoroughly with DMF and then dried under a stream of N_2 gas. The solution of WRKY1N (10 µM in HEPES buffer, pH = 7.0) was dropped onto the devices with maleimide modification to react for 12 h at 4 °C. PMMA was lifted off by using acetone. After rinsed with HEPES buffer (pH = 7.0) and dried with a stream of N_2 gas, the device was characterized by using AFM (Bruker AFM Dimension Icon). The AFM image (Figure R2) was generated at ScanAsyst mode with a sampling rate of 1.30 Hz and 256 samples per line, as shown in Figure S8, which confirmed the presence of a single protein attached on the side of silicon nanowire.

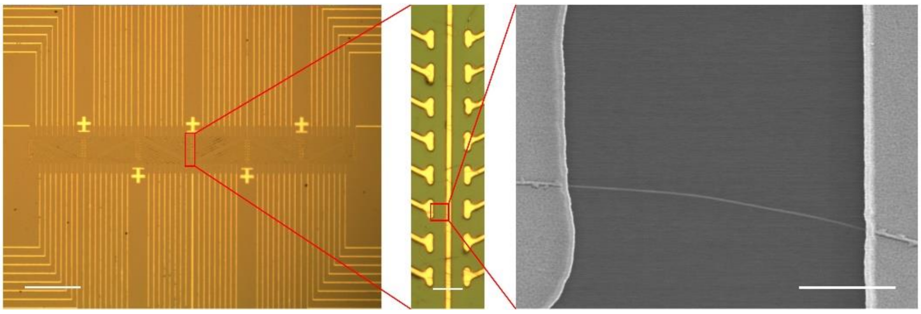

Figure S6. Optical and SEM images of a SiNW FET array. Left and medium images were taken by Nikon Eclipse LV 100 under 20x and 50x objectives. The right SEM image was generated by Hitachi S-4800. The scale bars from left to right are 300 µm, 10 µm and 2 µm, respectively.

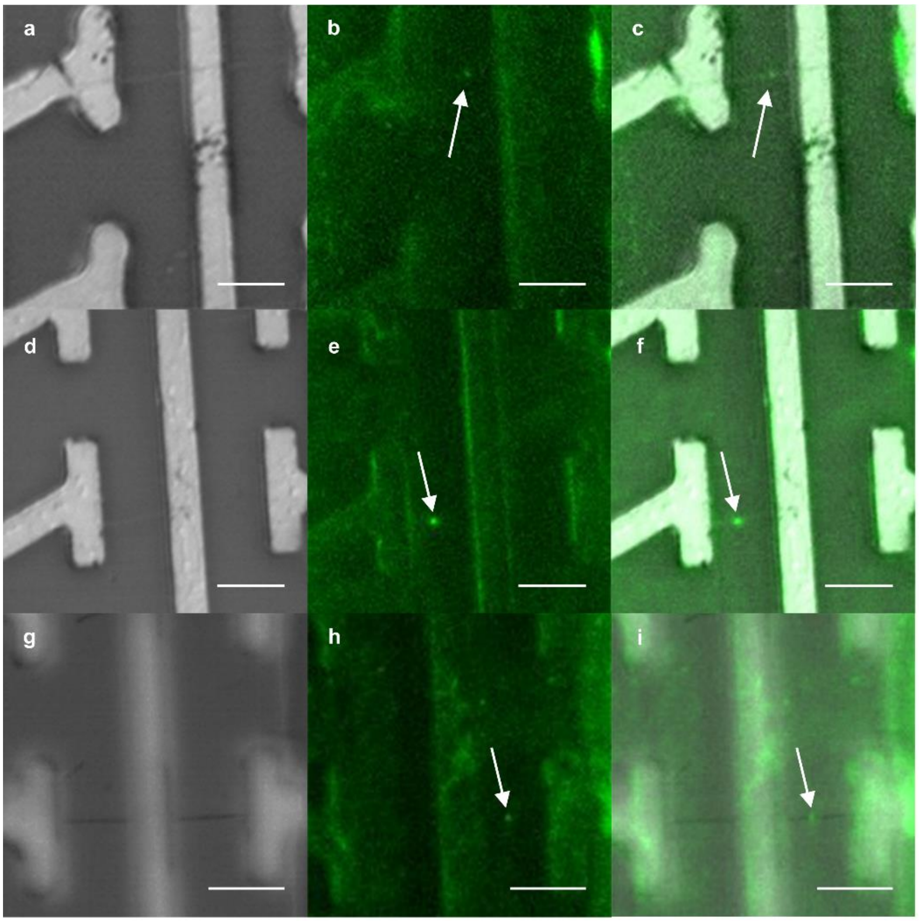

Figure S7. Characterization of single-DNA-modified SiNW FET devices. a, d, g) Photos of SiNW devices in the bright field. Only a single SiNW locates between two electrodes. b, e, h) Images of single-DNA-modified devices in the dark field. The photos are taken under the excitation light of 561 nm wavelength. c, f, i) Corresponding merged images in the bright and dark fields. The single fluorescence point was observed on the SiNWs between electrodes. Scale bar: 5 μm.

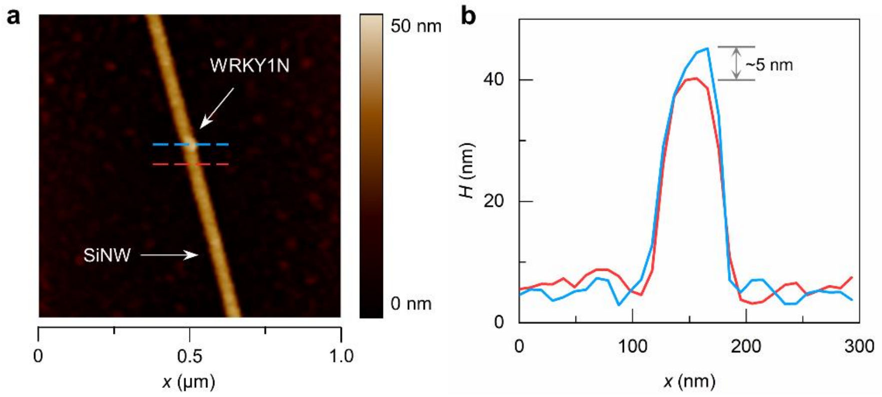

Figure S8. AFM image of a single WRK1N modified SiNW-FET device. a) AFM image of a SiNW device, where a single WRKY1N protein is attached on the surface of SiNW. b) Height distribution of different sections in the AFM image (red for a bare SiNW and blue for the locus of protein attachment). The difference of the height is about 5 nm, which is consistent with the size of WRKY1N (~4 nm) and the molecular linkage (~1 nm).

S3. Real-time current measurement and dynamic analysis

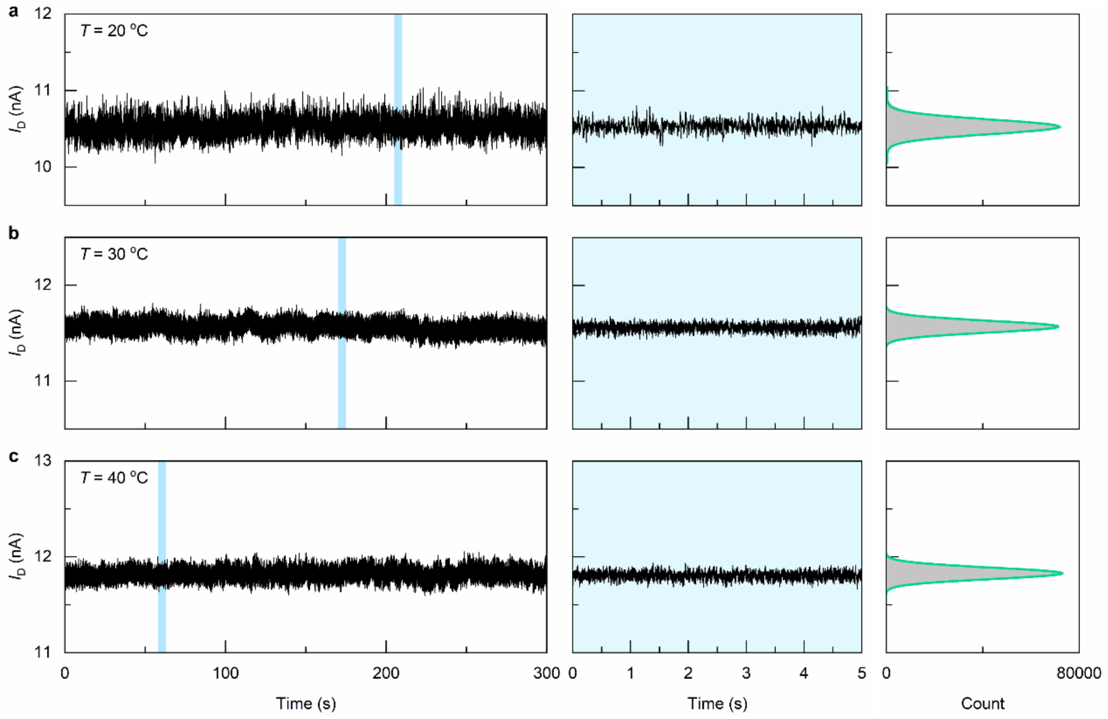

Figure S9. Control experiments of a DNA-modified device in the blank buffer. a–c) These graphs show 5-minute data sets of a representative single DNA-modified device carried out in a HEPES buffer solution (100 mmol L^{-1} NaCl) at 3 different temperatures: 20 °C (a), 30 °C (b) and 40 °C (c). The left panels of each graphs show real-time current recordings, the right panels are the corresponding current histograms and the medium panels are 5-second magnified view of current recordings (marked with blue columns). There are no significant changes in current flow through the devices except for noise signals.

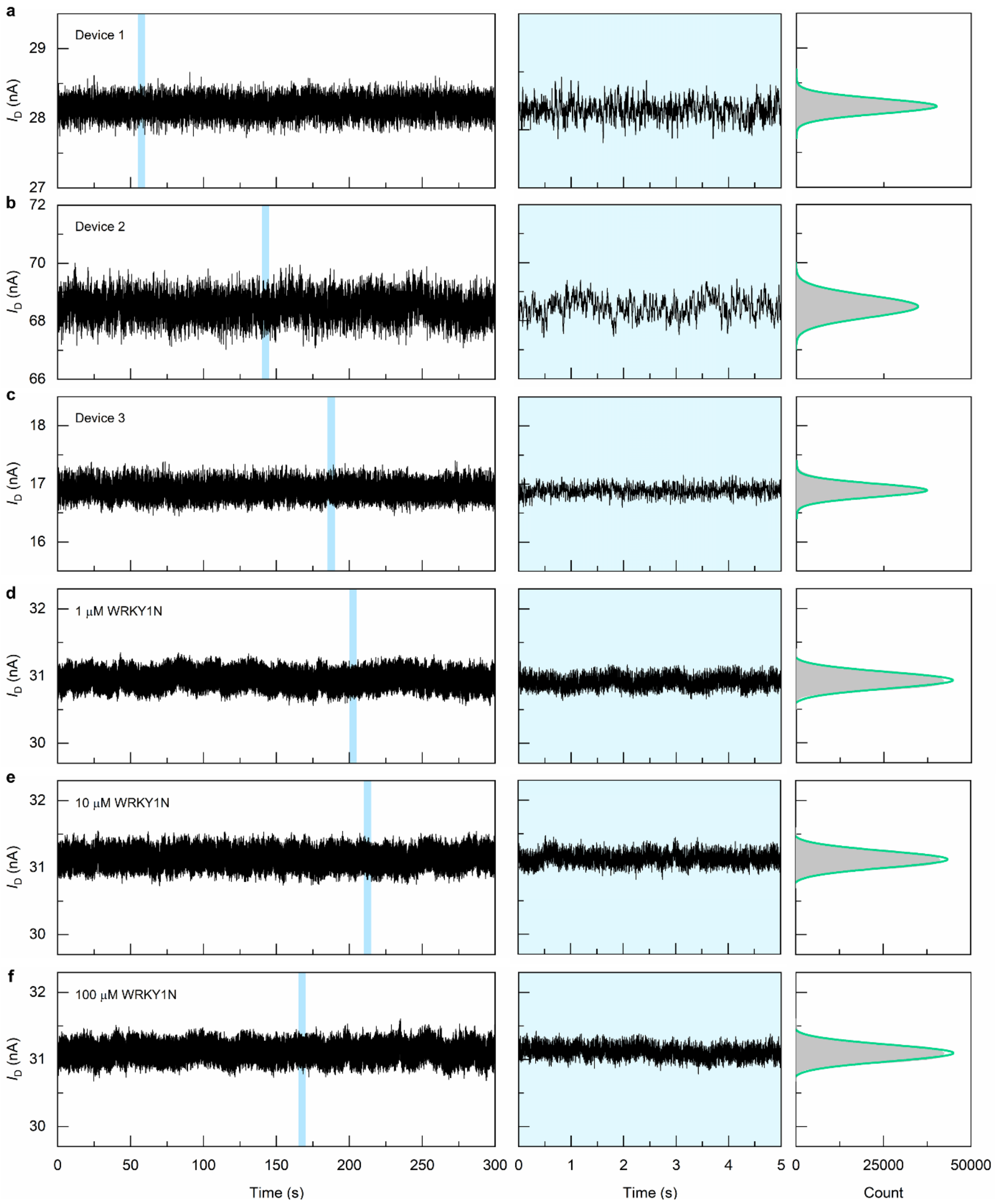

Figure S10. Control experiments of bare SiNW devices in the WRKY1N solution. a–c) These graphs show 5-minute data sets of 3 representative single-DNA-modified devices (a, Device 1; b, Device 2; c, Device 3) carried out in a WRKY1N solution (10 mmol L^{-1} HEPES, 100 mmol L^{-1} NaCl, 10 μmol L^{-1} WRKY1N) at 20 °C. e–f) 5-minute data sets of a single-DNA-modified device in WRKY1N solution of different concentrations (d, 1 μmol L^{-1}; e, 10 μmol L^{-1}; f, 100 μmol L^{-1}). The left panels of each graphs show real-time current recordings, the right panels are the corresponding current histograms and the medium panels show 5-second magnified view of current recordings (marked with blue columns). There are no significant changes in current flow through the devices except for noise signals.

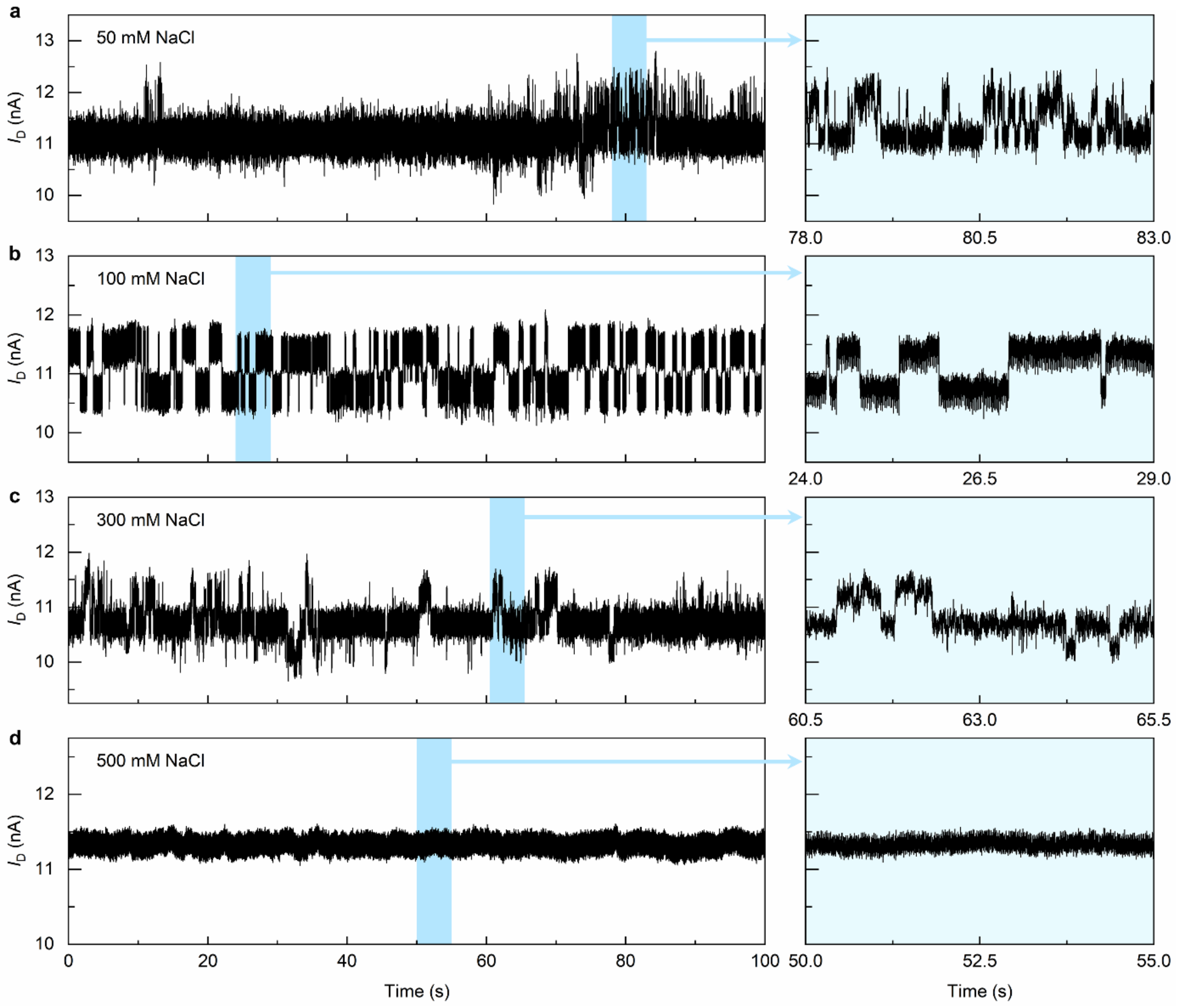

Figure S11. Salt concentration-dependent experiments. Real-time data of the DNA-modified device in a WRKY1N solution with different salt (NaCl) concentrations. The left panels show 100-second data of F1 DNA with different salt concentrations measured at 25 °C in a WRKY1N (10 μmol L^{-1}) HEPES (10 mM) solution from 50 mmol L^{-1} (a), 100 mmol L^{-1} (b), 300 mmol L^{-1} (c) and 500 mmol L^{-1} (d), respectively. The right panels are the 10-second magnified views of blue marked regions in the left panels.

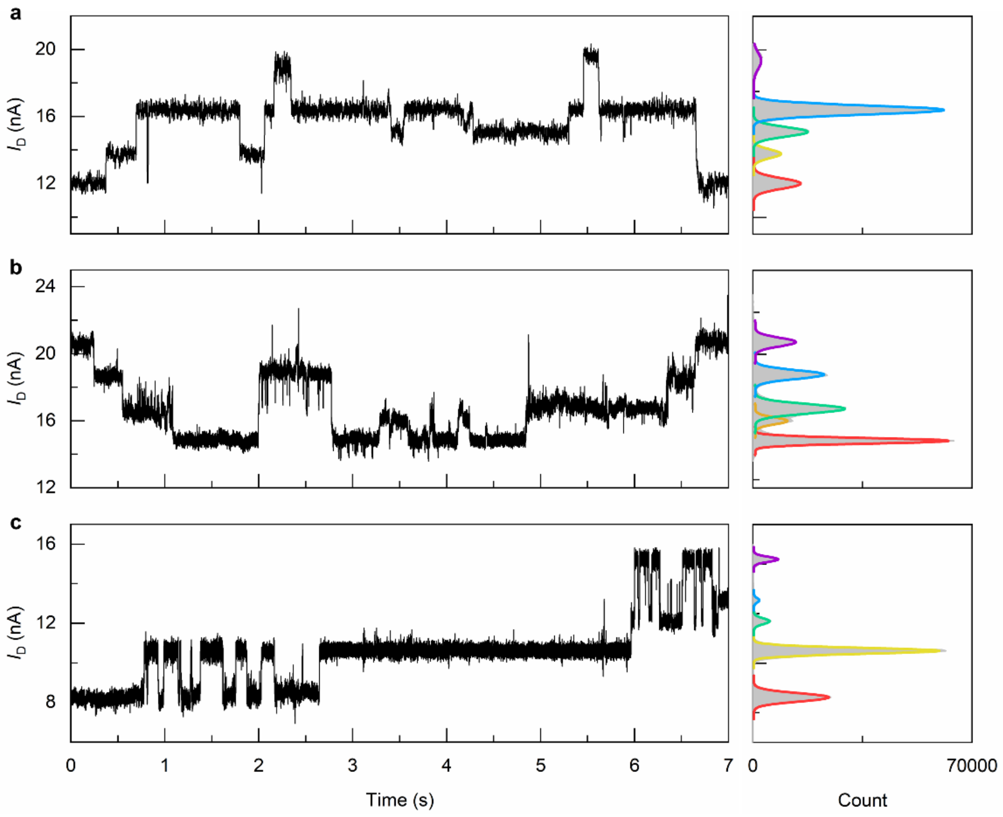

Figure S12. Slow multi-step signals of different devices. a–c) These graphs show 30-s data sets of 3 different single-DNA-modified devices in a WT-WRKY1N solution (100 mmol L^{-1} NaCl, 10 μmol L^{-1} WRKY1N) at 25 °C: F1-DNA (a), F4-DNA (b) and F5-DNA (c). Multi-stage signals appear in all experiments with these three single-DNA-modified devices.

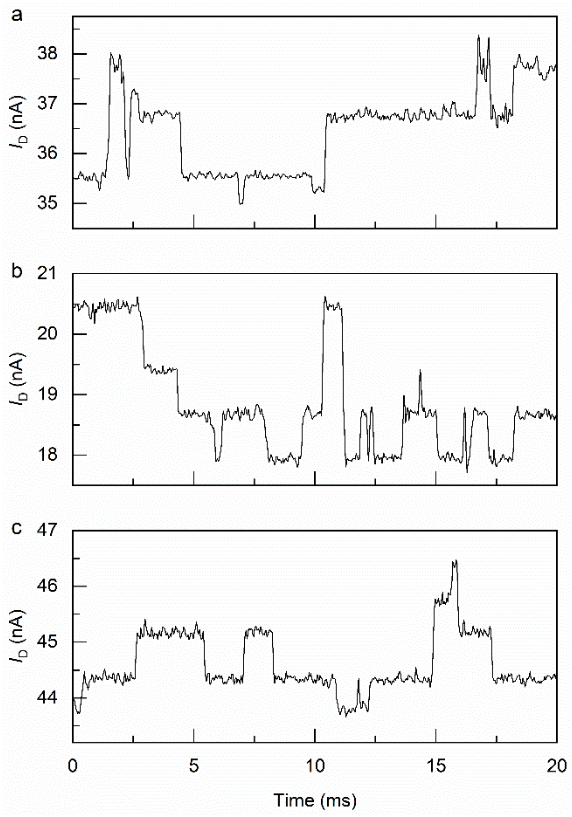

Figure S13. Fast multi-step signals of different devices. a–c, Real time trajectories of fast multi-step signals from different devices at 25 ^{o}C. The data was collected at a sampling rate of 28800 Sa s^{-1} (~34 μs interval).

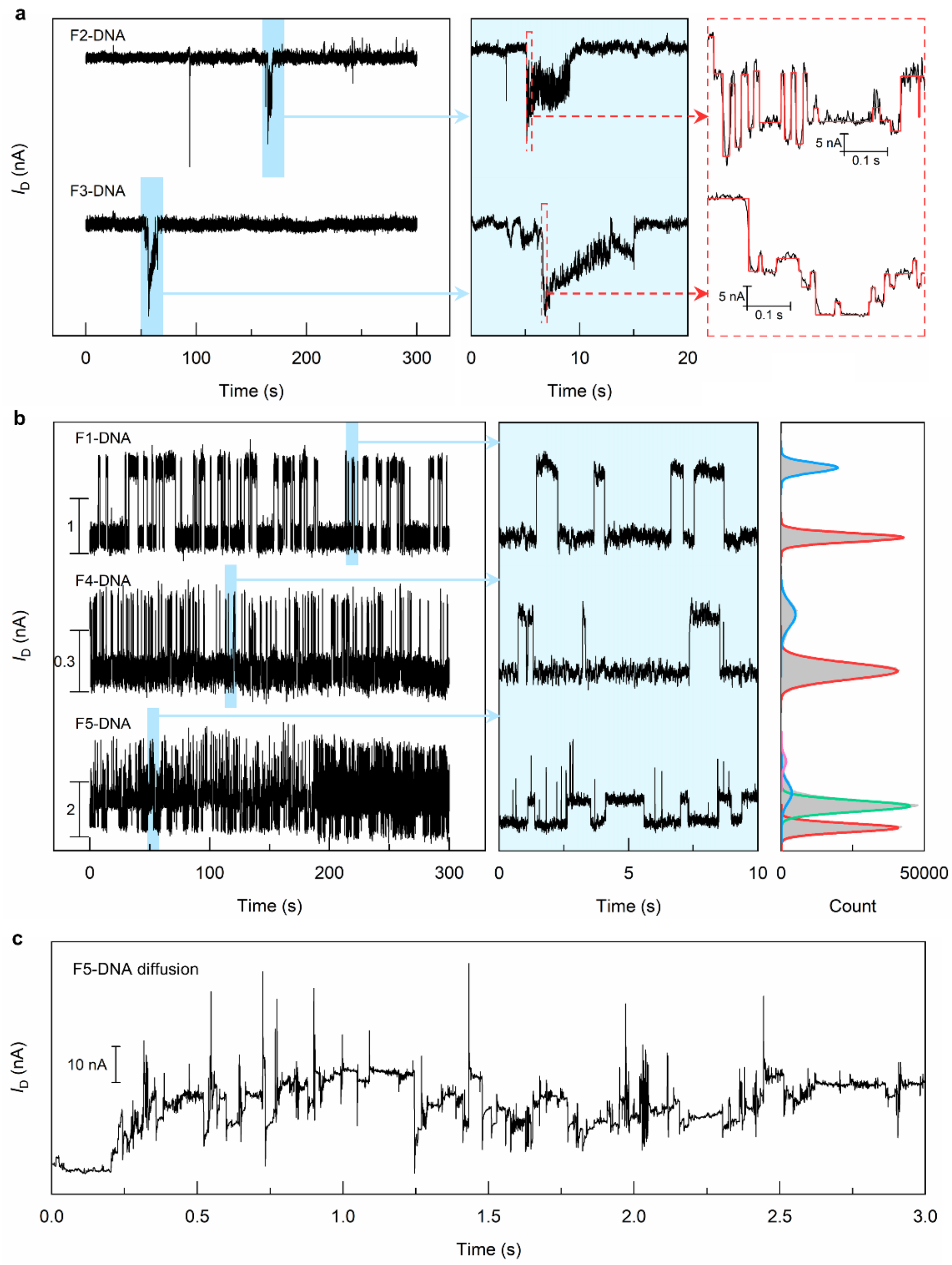

Figure S14. Measurements of WRKY1N protein interaction with different DNAs. a) The left panels show a 300-second real-time data of WRKY1N interacting with non-specific DNA F2-DNA (random sequence) and F3-DNA (2 bases mutated from F1-DNA). The medium panels are the magnified views of 20-second data (marked with blue columns) and the right red dash boxes show the detailed multistep signals within 0.5 s (magnified from the red dash box in the medium panels, black for raw data, red for idealized data). b) The left panel shows a 300-second real-time data of different length DNA with a single cognate binding site interacting with WRKY1N. The data from a and b were recorded at 25 °C in a 10 μmol L^{-1} WRKY1N solution. The medium panel shows the 10-second magnified views. The right panels show the corresponding current histograms. c) Measurements of WRKY1N protein diffusion along F5-DNA. The graph shows a 3-second current trajectory.

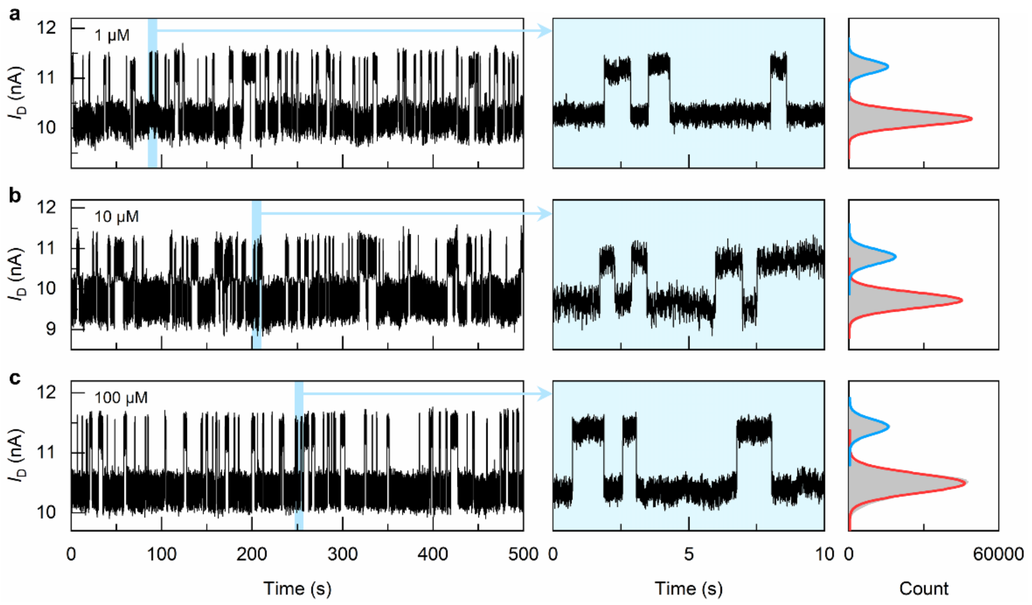

Figure S15. Measurements of WRKY1N concentration-dependent experiments. Real-time data of the specific WRKY1N-DNA binding process with different WRKY1N concentrations. The left panel shows 10-minute data of F1 DNA with different WRKY1N concentrations measured at 20 ^{o}C in a HEPES buffer solution from 1 μmol L^{-1} (a), 10 μmol L^{-1} (b) to 100 μmol L^{-1} (c), respectively. The medium panels are the 10-second magnified view and the right panels are the corresponding current histograms.

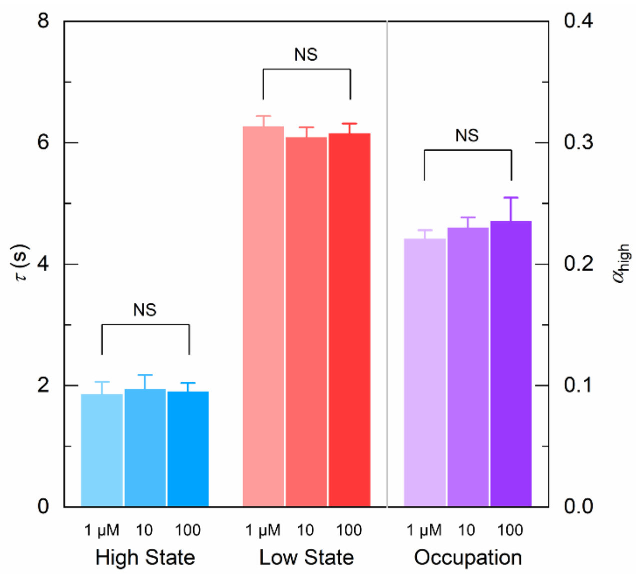

Figure S16. Comparison of signal properties in different WRKY1N concentrations (1 μM, 10 μM and 100 μM, n = 3). Both high (blue columns) and low (red columns) states have no significant differences in kinetic properties. The occupation of either high or low states (purple columns) has not significant differences when the WRKY1N concentration changes, indicating the unchanged thermodynamic properties. Error bars denote standard deviations, showing not significant differences ($P > 0.05$) by one-way ANOVA test. NS: not significant different.

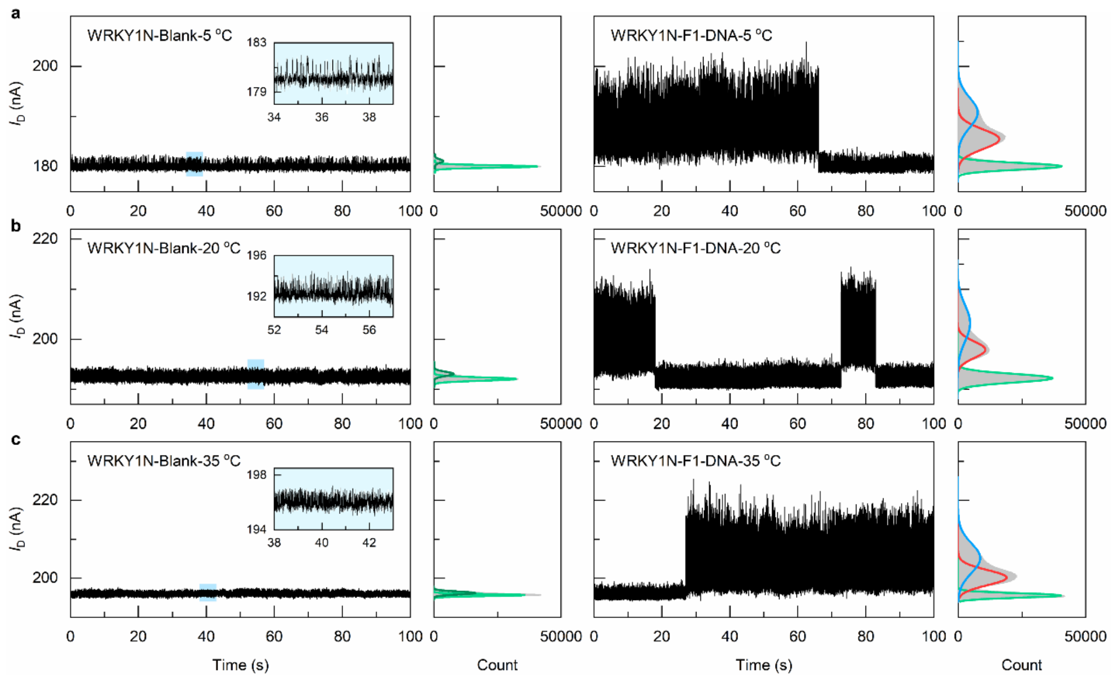

Figure S17. Temperature-dependent experiments of the WRKY1N-modified device. These experiments were carried out in a HEPES buffer (10 mmol L^{-1} HEPES, 100 mmol L^{-1} NaCl, pH = 7) or a F1-DNA solution (10 μmol L^{-1} F1-DNA, 10 mmol L^{-1} HEPES, 100 mmol L^{-1} NaCl, pH = 7) at 3 different temperatures: 5 °C (a), 20 °C (b) and 35 °C (c). *I*-*t* curves in each graph show real-time recordings of the current signals at each temperature. Histograms show the current distribution at each temperature. The insets show the magnified views of the marked area of the current recording (marked with blue rectangles). Small fluctuations appeared when the device was tested in the blank buffer and became negligible when the device was tested in the DNA solution, which means that the weak current change caused by WRKY1N protein is not the reason for the appearance of the two-state oscillation.

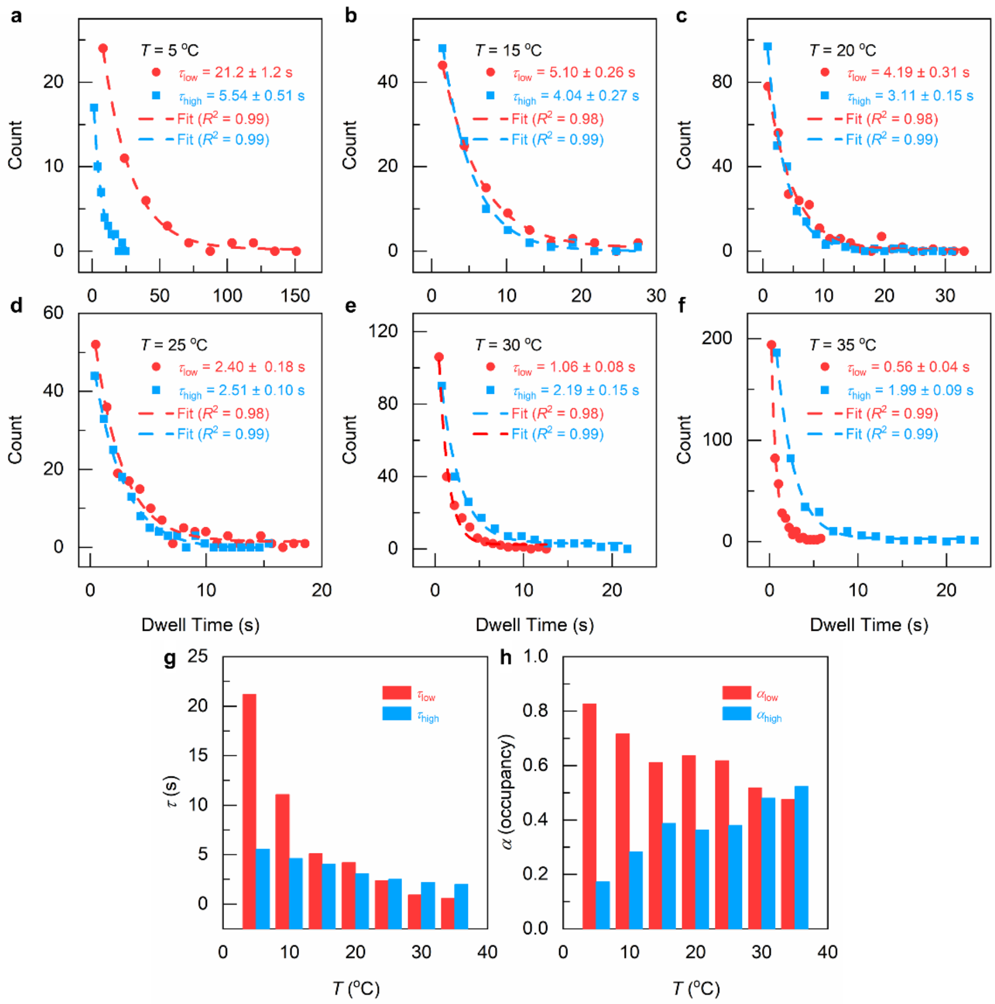

Figure S18. Analysis of temperature-dependent experiments (10 μmol L^{-1} WRKY1N). a–f) The graphs display the dwell-time distributions of a single DNA-modified device at different temperatures: 5 °C (a), 15 °C (b), 20 °C (c), 25 °C (d), 30 °C (e) and 35 °C (f). The distributions of both the low (red) and high (blue) states show a single exponential decay fits, generating the average dwell times, τ_{low} and τ_{high}. g) Dwell times of low and high states at each temperature. h) The occupancy (α) of high and low states at each temperature. The high state becomes predominant when temperature reaches 35 °C.

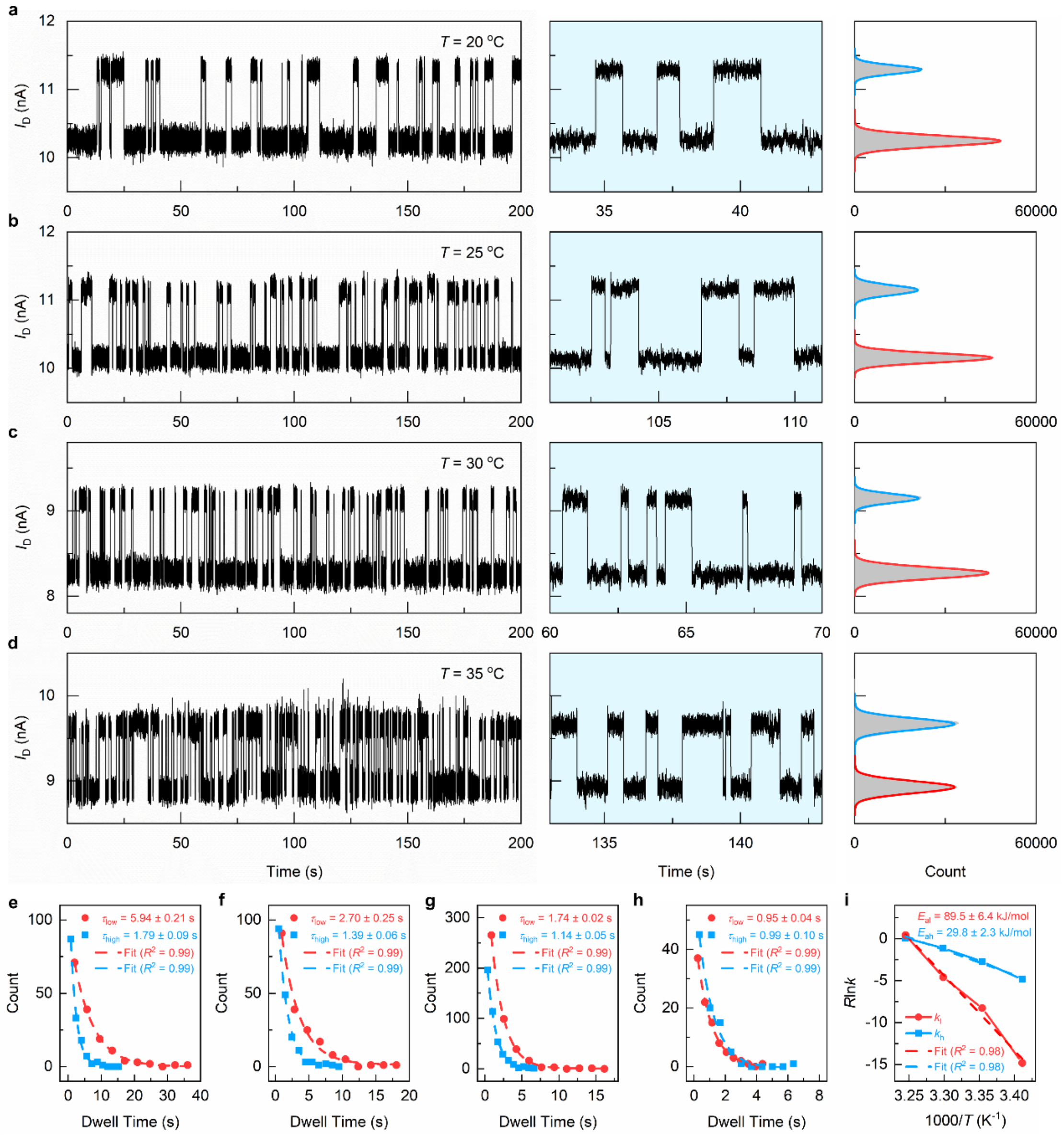

Figure S19. Analysis of temperature-dependent experiments (100 μmol L^{-1} WRKY1N). a–d) The experiments were carried out in a WRKY1N solution (100 μmol L^{-1} WRKY1N, 10 m mol L^{-1} HEPES, 100 m mol L^{-1} NaCl) at 4 different temperatures: 20 °C (a), 25 °C (b), 30 °C (c) and 35 °C (d). The left panel of each graph shows 200-second real-time current recordings, the right panels are the corresponding current histograms and the medium panels show the 10-second magnified data of each current recording. e–h) The distribution of the dwell times for high and low current states at 20 °C (e), 25 °C (f), 30 °C (g) and 35 °C (h). Dash lines represent the single-exponential fitting of the distributions (red for low state and blue for high state). i) Arrhenius plots of the signal changes between two different current states (high state in blue and low states in red) of the specific binding of WRKY1N and the corresponding DNA.

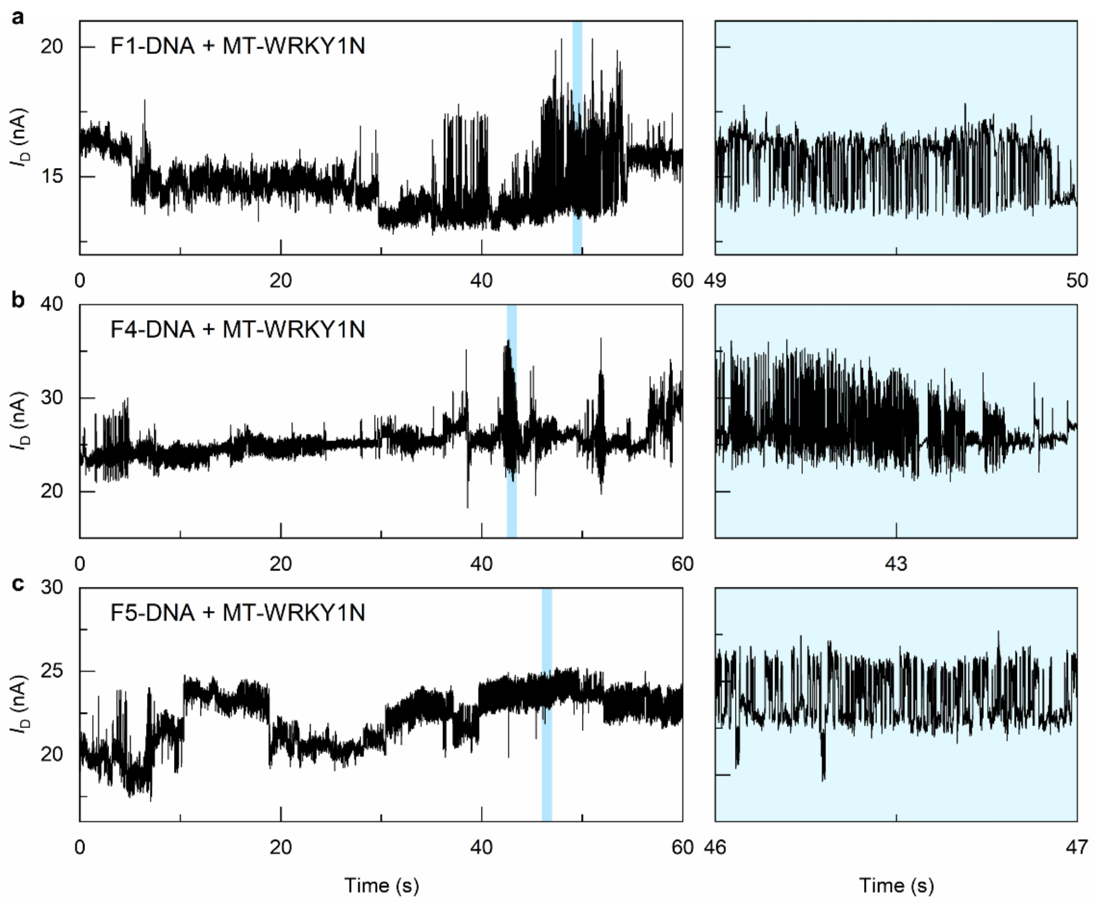

Figure S20. Experiments of mutant WRKY1N (MT-WRKY1N). These graphs show 1-minute data sets of 3 different single-DNA-modified devices in a MT-WRKY1N solution (100 mmol L^{-1} NaCl, 10 μmol L^{-1} MT-WRKY1N) at 25 °C: F1-DNA (a), F4-DNA (b) and F5-DNA (c). The right panels of these graphs show 1-minute current recordings and the left panels show the 1-second magnified view of the blue marked areas.

Table S1. DNA sequence information.

DNA	Sequence (5'-3') [a)]	Note
F1	GCGCTGGTCAAAGGCG	Single binding site
F2	GCGCTCTCGAGAGGCG	Nonspecific
F3	GCGCTGATTAAAGGCG	Mutation from F1
F4	CTGGTCAAAG	Shorter sequence (10 nt)
F5	ACCTTATCGCGCTGGTCAAACGCGATATAGAA	Longer sequence (32 nt)

[a)] Specific sequences are shown in red and mutation bases are shown in red.

Table S2. Kinetic and thermodynamic analysis of concentration-dependent experiments.

Parameter	Conc. of WRKY1N [μmol L^{-1}] [a)]		
	1	10	100
τ_{low} [s]	6.28 ± 0.16	6.11 ± 0.15	6.18 ± 0.14
τ_{high} [s]	1.88 ± 0.19	1.96 ± 0.22	1.92 ± 0.12
k_{low} [s^{-1}]	0.159 ± 0.004	0.164 ± 0.004	0.162 ± 0.004
k_{high} [s^{-1}]	0.53 ± 0.05	0.51 ± 0.06	0.52 ± 0.03
ΔI [nA]	1.045 ± 0.015	1.047 ± 0.030	1.047 ± 0.017
α_{high} [%]	22.2 ± 0.6	23.1 ± 0.7	23.6 ± 1.8
α_{low} [%]	77.8 ± 0.6	76.9 ± 0.8	76.4 ± 1.8

[a)]Data representation mean ± SD, sample size n = 3.

References:

[1] Y. P. Xu, H. Xu, B. Wang, X. D. Su, *Protein Cell* **2020**, *11*, 208.

[2] F. Patolsky, G. F. Zheng, C. M. Lieber, *Nat. Protoc.* **2006**, *1*, 1711.

[3] J. D. Wang, F. X. Shen, Z. X. Wang, G. He, J. W. Qin, N. Y. Cheng, M. S. Yao, L. D. Li, X. F. Guo, *Angew. Chem. Int. Ed.* **2014**, *53*, 5038.

[4] J. Li, G. He, H. Ueno, C. Jia, H. Noji, C. Qi, X. Guo, *Nanoscale* **2016**, *8*, 16172.

[5] J. Li, G. He, U. Hiroshi, W. Liu, H. Noji, C. Qi, X. Guo, *ACS nano* **2017**, *11*, 12789.

[6] Z. H. Yang, C. H. Qi, W. Z. Liu, D. B. Yin, L. J. Yu, L. D. Li, X. F. Guo, *Journal of Physical Chemistry Letters* **2021**, *12*, 3853.

[7] G. He, J. Li, H. N. Ci, C. M. Qi, X. F. Guo, *Angew. Chem. Int. Ed.* **2016**, *55*, 9036.
